# Deep multi-region whole-genome sequencing reveals heterogeneity and gene-by-environment interactions in treatment-naive, metastatic lung cancer

**DOI:** 10.1038/s41388-018-0536-1

**Published:** 2018-10-22

**Authors:** Tracy L. Leong, Velimir Gayevskiy, Daniel P. Steinfort, Marc R. De Massy, Alvaro Gonzalez-Rajal, Kieren D. Marini, Emily Stone, Venessa Chin, Adrian Havryk, Marshall Plit, Louis B. Irving, Barton R. Jennings, Rachael A. McCloy, W. Samantha N. Jayasekara, Muhammad Alamgeer, Vishal Boolell, Andrew Field, Prudence A. Russell, Beena Kumar, Daniel J. Gough, Anette Szczepny, Vinod Ganju, Fernando J. Rossello, Jason E. Cain, Anthony T. Papenfuss, Marie-Liesse Asselin-Labat, Mark J. Cowley, D. Neil Watkins

**Affiliations:** 1 ACRF Stem Cells and Cancer Division, The Walter and Eliza Hall Institute of Medical Research, Parkville, VIC 3050 Australia; 2 Department of Medical Biology, University of Melbourne, Parkville, VIC 3050 Australia; 3 The Kinghorn Centre for Clinical Genomics, Garvan Institute of Medical Research, Darlinghurst, NSW 2010 Australia; 4 Department of Respiratory Medicine, Royal Melbourne Hospital, Parkville, VIC 3050 Australia; 5 The Kinghorn Cancer Centre, Garvan Institute of Medical Research, Darlinghurst, NSW 2010 Australia; 6 The Hudson Institute of Medical Research, Clayton, VIC 3168 Australia; 7 Department of Thoracic Medicine, St Vincent’s Hospital, Darlinghurst, NSW 2010 Australia; 8 St Vincent’s Clinical School, UNSW Sydney, Darlinghurst, NSW 2010 Australia; 9 Department of Medical Oncology, St Vincent’s Hospital, Darlinghurst, NSW 2010 Australia; 10 Department of Respiratory and Sleep Medicine, Monash Health, Clayton, VIC 3168 Australia; 11 Department of Pathology, St Vincent’s Hospital, Sydney, NSW 2010 Australia; 12 Department of Pathology, St Vincent’s Hospital Melbourne, Fitzroy, VIC 3000 Australia; 13 Department of Pathology, Monash Health, Clayton, VIC 3168 Australia; 14 Department of Molecular and Translational Science, Faculty of Medicine, Nursing and Health Sciences, Monash University, Clayton, VIC Australia; 15 Department of Anatomy and Developmental Biology, Faculty of Medicine, Nursing and Health Sciences, Monash University, Clayton, VIC 3168 Australia; 16 Australian Regenerative Medicine Institute, Monash University, Clayton, VIC 3168 Australia; 17 Computational Cancer Biology Program, Peter MacCallum Cancer Centre, Melbourne, VIC 3000 Australia; 18 Sir Peter MacCallum Department of Oncology, The University of Melbourne, Parkville, VIC 3052 Australia; 19 Bioinformatics Division, The Walter & Eliza Hall Institute of Medical Research, Parkville, VIC 3052 Australia; 20 Children’s Cancer Institute, Kensington, NSW 2750 Australia

**Keywords:** Non-small-cell lung cancer, Small-cell lung cancer, Cancer genomics

## Abstract

Our understanding of genomic heterogeneity in lung cancer is largely based on the analysis of early-stage surgical specimens. Here we used endoscopic sampling of paired primary and intrathoracic metastatic tumors from 11 lung cancer patients to map genomic heterogeneity inoperable lung cancer with deep whole-genome sequencing. Intra-patient heterogeneity in driver or targetable mutations was predominantly in the form of copy number gain. Private mutation signatures, including patterns consistent with defects in homologous recombination, were highly variable both within and between patients. Irrespective of histotype, we observed a smaller than expected number of private mutations, suggesting that ancestral clones accumulated large mutation burdens immediately prior to metastasis. Single-region whole-genome sequencing of from 20 patients showed that tumors in ever-smokers with the strongest tobacco signatures were associated with germline variants in genes implicated in the repair of cigarette-induced DNA damage. Our results suggest that lung cancer precursors in ever-smokers accumulate large numbers of mutations prior to the formation of frank malignancy followed by rapid metastatic spread. In advanced lung cancer, germline variants in DNA repair genes may interact with the airway environment to influence the pattern of founder mutations, whereas similar interactions with the tumor microenvironment may play a role in the acquisition of mutations following metastasis.

## Introduction

Genomic heterogeneity is now recognized as a major challenge to the success of conventional therapy, targeted therapy, and immunotherapy in the treatment of cancer [1]. The advent of multi-region sequencing has led to the identification of a previously unknown degree of complexity and genomic heterogeneity in solid tumors [2]. These landmark findings have major implications for the understanding of tumor initiation and evolution, particularly with respect to the development of metastasis [3]. Furthermore, they illustrate how analyzing multiple tumor sites from a relatively small number of cases can reveal the complexities of genomic evolution that cannot be resolved by single-site sequencing of large cohorts [1].

Although conclusions vary in different tumor systems, the use of genomic data to model tumor phylogenies suggests that metastasis can occur early in the life of the primary tumor [4]. There is also debate as to whether the emergence of private mutations in metastatic disease drives metastasis from rare clones present in the primary tumor, are the result of local environmental pressures unique to each metastatic site, or are random passenger events [5]. The propensity of lung cancer to spread to local and regional lymph nodes or beyond is reflected in the small numbers of patients eligible for curative surgery, and the lack of success in treating inoperable, locally advanced tumors with radical chemo-radiotherapy. Lung cancer represents a major challenge with respect to mapping genomic heterogeneity, in that most patients are inoperable at diagnosis [6]. Since surgical specimens offer high-quality tissue samples, early-stage lung cancers have largely formed the basis of large-scale sequencing projects in lung cancer [7–9], a better understanding of genomic heterogeneity in advanced lung cancer may inform the successful deployment of targeted therapy and immunotherapy.

Reflecting these technical barriers, multi-region sequencing analysis of lung cancer has been largely limited to surgically resected specimens [10, 11]. In this regard, access to high-quality specimens from metastatic lymph nodes in surgical cases is limited, since the entire node is required for accurate diagnosis and staging rather than research. Therefore, the degree to which genomic heterogeneity might contribute to the pathogenesis of treatment-naive, metastatic lung cancer remains an open question. To address this uncertainty, we combined two approaches that circumvented the need to rely on surgical specimens. First, we obtained samples from newly diagnosed lung cancer patients undergoing endobronchial ultrasound-guided transbronchial needle aspiration (EBUS-TBNA), a minimally invasive technique that yields high-quality samples from both the primary tumor and intrathoracic lymph nodes in the same patient [12]. Second, we analyzed these specimens using high-depth whole-genome sequencing (WGS), which provides advantages over whole-exome sequencing (WES) in cancer specimens with respect to uniformity of coverage, high-resolution detection of copy number variation (CNV) and structural variation (SV) [13, 14], as well as genome-wide, unbiased assessment of somatic mutation signatures [15]. Here we apply the principles of multi-region sequencing to metastatic lung adenocarcinoma (LUAD), squamous cell carcinoma (LUSC), and small cell lung cancer (SCLC), the three commonest forms of the disease.

## Results

### Intra-patient heterogeneity in metastatic lung cancer

**Fig. 1:**
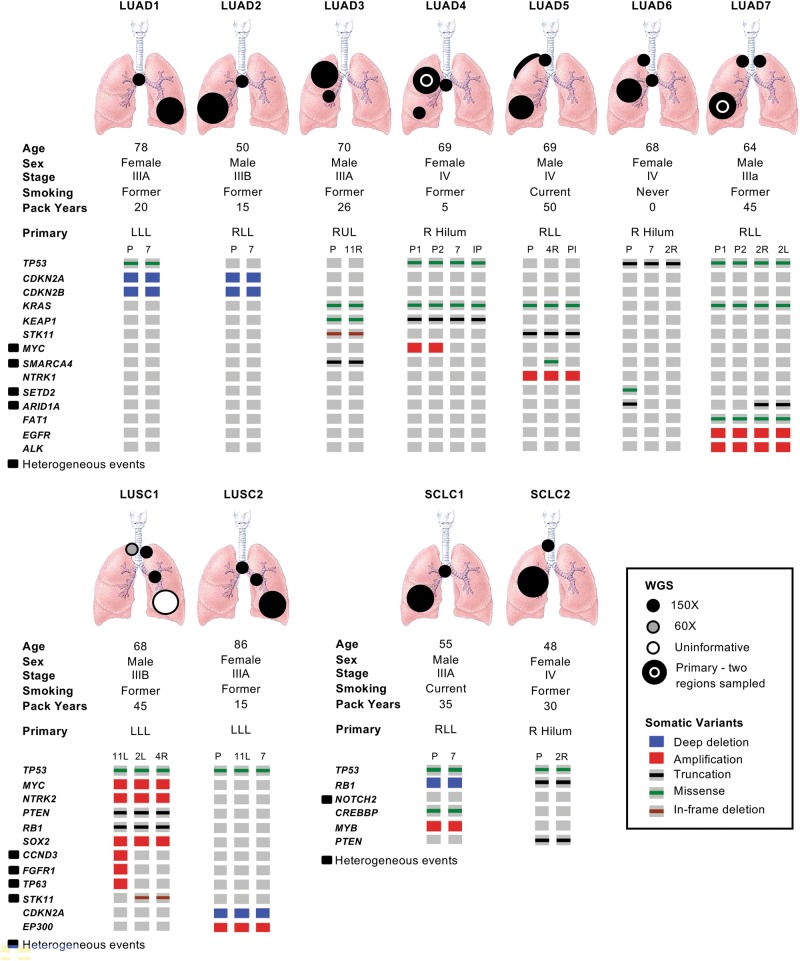
Overview of cases analyzed with multi-region whole-genome sequencing. Oncoprint data depicting single-nucleotide variants, insertion/deletions, and copy number events in known lung cancer driver genes for each sample are shown with heterogeneous mutations labeled. LUAD lung adenocarcinoma, LUSC lung squamous cell carcinoma, SCLC small cell lung cancer, P primary tumor, IP intrapulmonary, Pl pleural. Metastases are otherwise indicated by lymph node station

A total of 30 primary and metastatic samples from 11 patients undergoing EBUS-TBNA for the diagnosis and/or staging of lung cancer were deemed informative based on tumor cellularity estimates of cytology smears and DNA quality (Fig. 1). Except for one sample, all were analyzed with WGS to an average depth of 150 × , including two cases in which two samples were obtained from the primary tumor in addition to the metastases. Peripheral blood mononuclear cells were used as a germline control sequenced to an average depth of 30–40 × . In addition, a further nine patients yielded informative samples from a single site and were sequenced to an average WGS depth of 60 × (Supplementary Figure S1).

When comparing primary with metastatic tumors, mutations in known lung cancer driver genes (Supplementary Data File S1) such as *TP53*, *KRAS*, *FAT1, PTEN*, and *RB1*, as well as homozygous deletions in *CDKN2A* and *RB1*, were conserved across all samples in individual patients (Fig. 1; Supplementary Data File S3). Exceptions included events private to the primary tumor (*SETD2* and *ARID1A* in LUAD6; *NOTCH2* in SCLC1) (Fig. 1), or private to metastases (*SMARCA4* in LUAD5; *ARID1A* in LUAD7) (Fig. 1). Heterogeneity was more prevalent with respect to copy number gain, with private amplification events in driver genes including *MYC, CCND3, FGFR1, and TP63* (Fig. 1; Supplementary Data File S3). Several of these amplification events were private to the primary tumor, rather than the associated metastasis (Fig. 1). This pattern of heterogeneity was similar when considering a broader list of pan-cancer driver genes (Supplementary Data File S1), with private amplification events seen in *ERBB3*, *RHEB*, *SOS1*, *SOS2*, and *EZH2* (Supplementary Figure S2).

We observed mutations in several important pan-cancer drivers not normally associated with lung cancer, including *TSC1* in LUAD1 and LUSC5, and *WT1* in LUAD6 and LUAD12. (Supplementary Figure S3). In addition, we detected medium and high-impact missense mutations associated with somatic loss-of-heterozygosity (LOH) in several novel genes with potential functional significance (Supplementary Table S1; Supplementary Figure S3). These included loss-of-function (LOF) variants in the Wilson’s disease copper exporter *ATP7B* (LUAD1), the Toll-like receptor *TLR4* (LUAD13), and in *ERAP2* (LUAD1), whose gene product promotes the presentation of endogenous cellular peptides by major histocompatibility complex class I molecules (Supplementary Table S1). Two potential gain-of-function (GOF) variants were also identified, one in the kinase domain of *JAK2* in LUAD4, the other in the growth factor receptor domain of *ERBB4* in LUSC4 (Supplementary Figure S3b).

Overall, these data are in keeping with similar results in metastatic pancreatic cancer [16], and suggest that there is limited heterogeneity in metastatic lung cancer with respect to mutations in key driver genes, and are consistent with a model in which these critical events are required for both the initiation and progression of the primary tumor prior to metastasis.

### Novel fusions

**Fig. 2:**
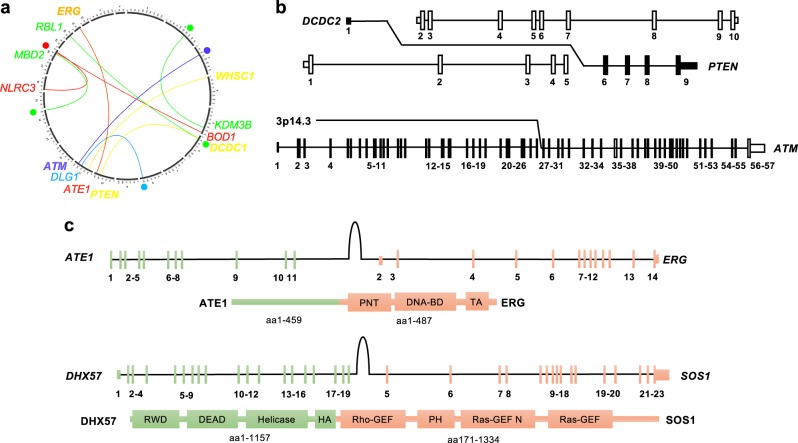
Novel fusion events. **a** Circos plot depicting examples of novel fusion events. Each case is represented by one color. Intergenic regions are indicted by colored circles. **b** Examples of candidate loss-of-function fusion events in the tumor-suppressor genes *PTEN* and *ATM*. **c** Models of candidate gain-of-function fusion events depicted at genome and protein level. Protein domains are labeled according to Pfam convention. Exons in each fusion partner are numbered

Fusions are now recognized as important GOF driver events in solid tumors, including well-described targetable events in genes such as *ALK*, *ROS1*, and *NTRK1* in LUAD [17]. However, LOF fusion events in solid tumors are less well characterized. In the 20 cases analyzed, we detected three fusion events in known cancer driver genes (*ATM* in LUSC2; *PTEN* in LUSC1; *WHSC1* in LUSC1), and a further seven events in candidate tumor-suppressor genes, including *BOD1*, *DLG2*, *MBD2*, and *RBL1* (Fig. 2a,b; Supplementary Figure S4; Supplementary Table S2). In each case, the predicted transcript resulted in truncated or nonsense gene products (Supplementary Table S2). In addition, we identified two candidate GOF fusion events (Fig. 2a,c; Supplementary Table S2; Supplementary Data File S3). In SCLC1, we detected a candidate fusion of the N terminus of the arginyltransferase *ATE1* to the pointed (PNT), DNA binding and transactivation domains of ERG, an ETS-family transcription factor subject to frequent GOF fusion events in prostate cancer [18]. In LUAD7, we detected a candidate fusion event involving the N terminus of the DEAH-Box Helicase DHX57 with the catalytic C terminus domain of the RAS guanine nucleotide exchange factor SOS1 (Fig. 2a,c). The detection of 12 novel fusions in only 20 cases of advanced lung cancer reflects the sensitivity of WGS in detecting such events, and suggests that pathogenic fusions may be much more common in advanced lung cancer than previously appreciated. The potential for fusions to inactivate critical tumor-suppressor genes is supported by the recent description of LOF rearrangements in *TP53* in osteosarcoma [19], and suggests that such events may add to mutation, deletion, and epigenetic gene silencing as an important path of tumor-suppressor inactivation.

### Somatic mutation signatures and germline variants in DNA repair genes

**Fig. 3:**
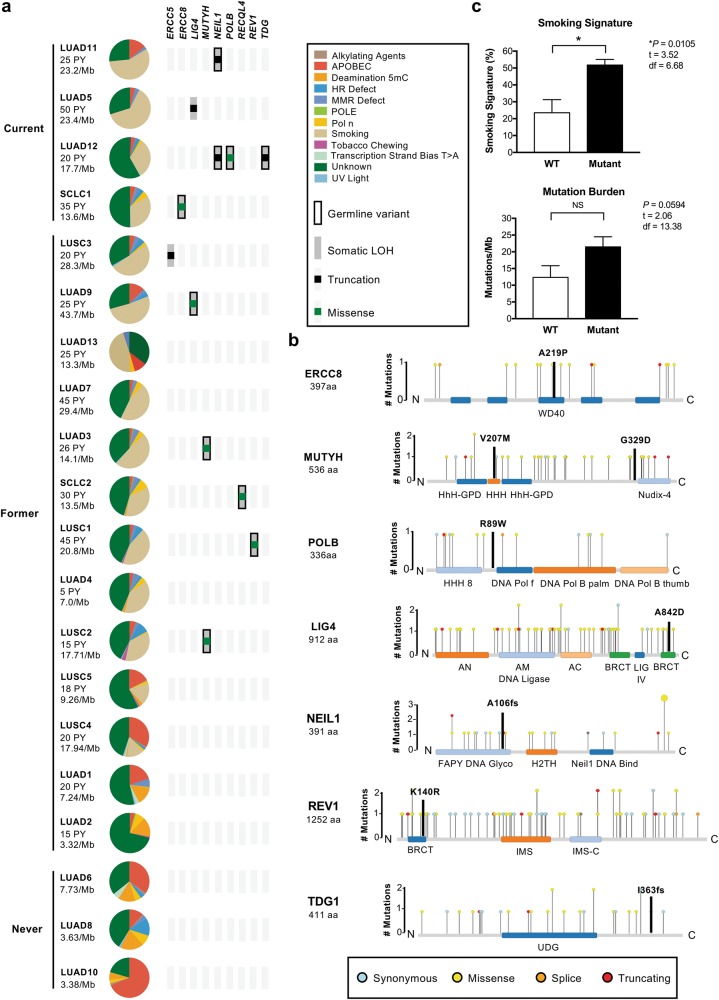
Germline and somatic mutations in DNA repair genes and somatic mutation signatures. **a** Cases are listed according to smoking status, then by the fraction of the somatic mutation burden attributable to smoking. Pack-year (PY) exposure and mutations per Mb are indicated. Variants in DNA repair genes are depicted in oncoprint format. **b** Examples of germline mutations in DNA repair genes associated with tumor loss-of-heterozygosity. Needle plots generated by IntOGen depict somatic mutations in cancer. Protein domains are indicated using Pfam nomenclature. **c** Quantification of smoking signature and tumor mutation burden in tumors from patients with wild type (WT, *n* *=* 6) or germline mutations (Mutant, *n* *=* 10) in the DNA repair genes shown in Fig. 3a. Welch’s *t*-test, two tailed. df degrees of freedom, NS not significant, LUAD lung adenocarcinoma, LUSC lung squamous cell carcinoma, SCLC small cell lung cancer

We compared the mutation burden and somatic signatures shared by all tumor samples in each multi-region case, and in the informative samples of single-site cases, with smoking history (Fig. 3a). As expected, tumors in the 4 current smokers, each with over 20 pack-years exposure, were hypermutated ( > 10 variants/Mb), and were dominated by a strong tobacco-related somatic signature (Fig. 3a). In keeping with published work, we also observed low mutation burdens in the tumors of the 3 never-smokers, which were dominated by Apolipoprotein B MRNA Editing Enzyme Catalytic Subunit 3G (APOBEC) and 5-methyl-cytosine (5-mC) deamination patterns typically associated with age or inflammation [20] (Fig. 3a). Tumors in the 13 former-smokers exhibited a wide range of mutation burdens (3.3–43.7/Mb) and smoking mutation signatures (0–57%), despite over 10 pack-years of exposure in all but one case (Fig. 3a). With the caveat that this observation is based on a small number of cases, this variation in mutation burden attributable to smoking was puzzling given the overwhelming evidence of a correlation between pack-year exposure and a smoking-related somatic signature in lung cancer [20]. Given the genotoxic effects of smoking on airway epithelial cells, and the importance of components of the DNA repair machinery in repairing this damage [21], we hypothesized that the degree of smoking-related DNA damage in each tumor may be related to germline variants in DNA repair genes.

We separately analyzed the germline WGS data from all 20 patients in the study without reference to the somatic mutations detected in the corresponding tumor samples. In 16 cases, we identified 30 potentially significant germline variants in DNA repair genes (Supplementary Table S3; Supplementary Data File S4). A subset of these genes is associated with repair of DNA damage induced by smoking, including *ERCC5*, *ERCC8*, *LIG4*, *MUTYH*, *NEIL1*, *POLB*, *RECQL4*, *REV1*, and *TDG* [21–23] (Fig. 3b; Supplementary Table S3). Although smoking can induce a range of genotoxic effects, these genes are of interest because they have been implicated in the repair of tobacco carcinogen adducts or oxidation-induced single-strand breaks. As shown in Fig. 3a, tumors with the strongest tobacco mutation signature occurred in patients with germline variants in these genes associated with somatic LOH. In addition, two hypermutated tumors with strong tobacco signatures harbored somatic mutations in *LIG4* (LUAD5) and *ERCC5* (LUSC3) (Fig. 3a). Considering all 17 ever-smokers as a group, the presence of a mutation in one or more of these genes was associated with a greater fraction of the somatic signature attributable to smoking (*P* < 0.05) but not overall mutation burden (Fig. 3c).

**Fig. 4:**
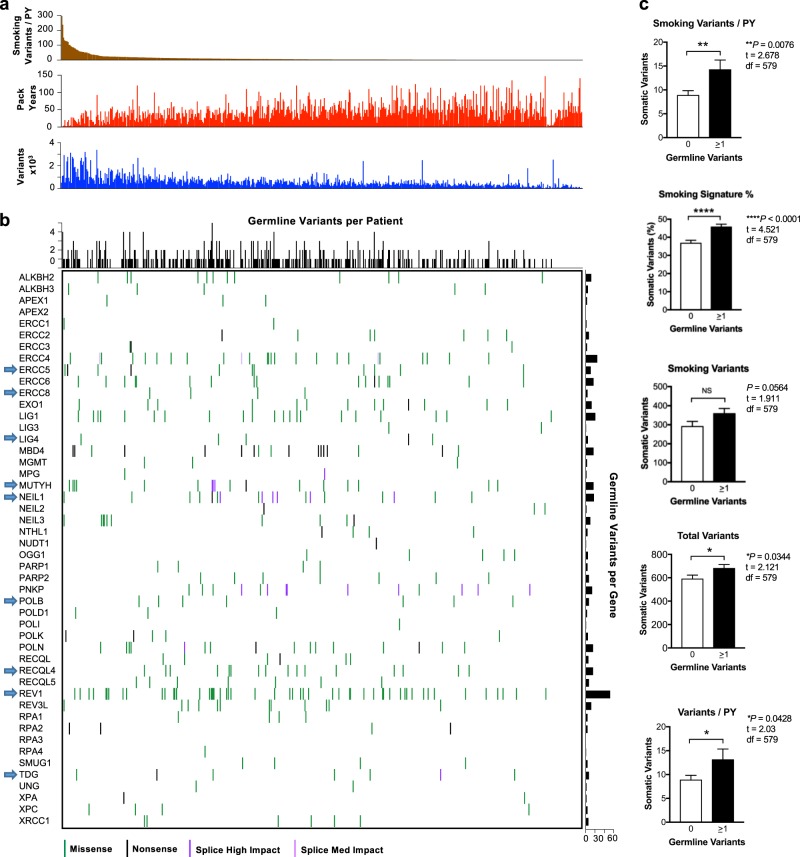
Analysis of somatic mutation patterns and germline variants of TCGA non-small cell lung cancer data. **a** Somatic mutation burden in cases arranged by the number of smoking signature variants per pack-year (PY) exposure. Matched data for each case indicating total pack-years and total somatic variants are shown directly below. **b** Heterozygous germline variants in DNA repair genes potentially linked to the repair of smoking-induced DNA damage arranged in the same order as above. Genes identified in the EBUS-TBNA cohort are labeled with an arrow. **c** Comparison of somatic mutation patterns between cases without germline variants (*n* = 314), or cases with one or more variants (*n* *=* 262). Mean ± SEM, unpaired *t*-test, two tailed. df degrees of freedom, NS not significant

To validate this observation, we analyzed 576 non-SCLC cases in TCGA dataset without oncogenic mutations in *EGFR* or *EML4-ALK* rearrangements in which pack-year cigarette exposure was available (Supplementary Data File S5). Neither somatic mutation burden, smoking somatic signature percentage, or the number of variants potentially caused by smoking correlated with smoking history (Supplementary Figure S5). Analysis of heterozygous germline events in 48 DNA repair genes likely to be associated with smoking-induced DNA damage (Supplementary Data File S2) identified 393 variants in 262 patients. Comparison of the estimated number of smoking-related variants per pack-year with these germline variants identified a group of patients with minimal smoking histories and large somatic tobacco mutation burdens with germline events in genes identified in our EBUS-TBNA cohort (*ERCC5*, *NEIL1*, *REV3L*, *REV1*), as well as the *NEIL1* ortholog *NEIL3* (Fig. 4). In the patient with the highest tobacco mutation burden per pack-year, we detected variants in four genes, *ERCC1*, *NEIL1*, *NEIL3*, and *REV3L* (Fig. 4). By contrast, we did not detect germline variants in a group of patients with very low somatic smoking burdens, despite a heavy smoking history (Fig. 4a, b). Consistent with our data in the EBUS-TBNA cohort, patients with one or more germline variants in this gene list had tumors with increased numbers of smoking variants per pack-year, and a higher percentage of somatic smoking signature (Fig. 4c). Rather than directly contributing to the risk of lung cancer, these data suggest that the smoking mutation signature may be the result of an interaction between damaging germline variants in specific DNA repair genes and cigarette exposure.

### Phylogenetic models of lung cancer metastasis

**Fig. 5:**
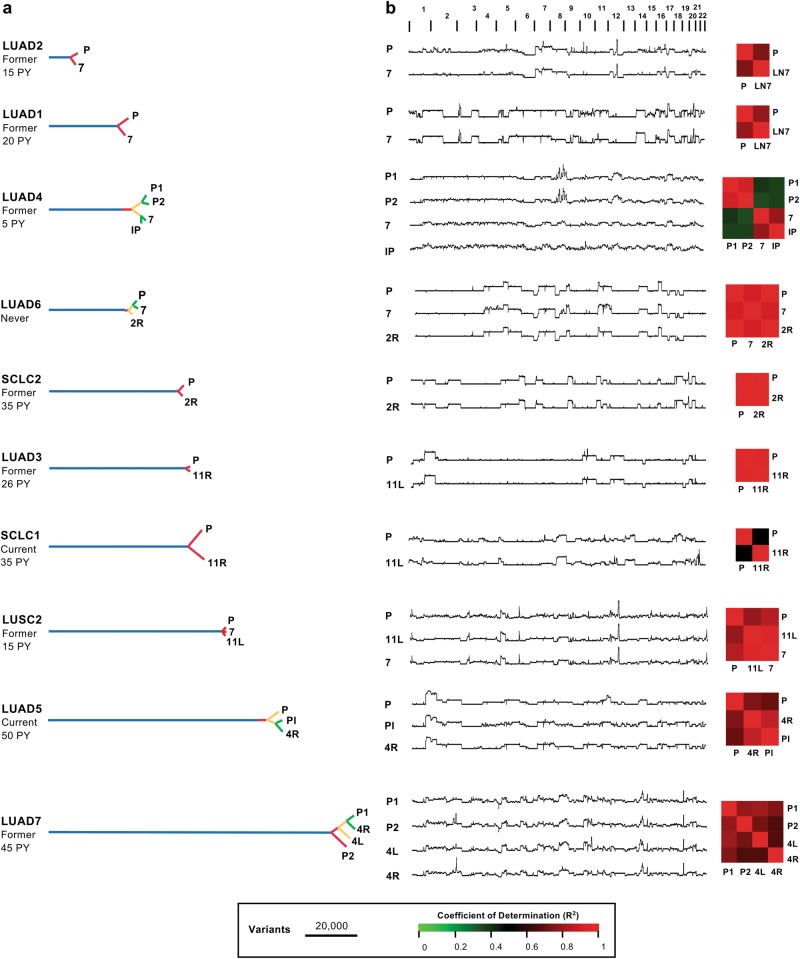
Progression model of lung cancers reconstructed from the analysis of shared and private variants. The smoking status and pack-year (PY) exposure for each case is listed. **a** SNVs and Indel counts shown to scale. **b** CNV plots scaled per patient for each sample. To the right, heat maps depict heterogeneity in copy number variation between samples in each case. P primary tumor; metastases are indicated by lymph node station except for Pl (pleura) and IP (intrapulmonary). LUAD lung adenocarcinoma, LUSC lung squamous cell carcinoma, SCLC small cell lung cancer

We prioritized resolving the degree of heterogeneity by focussing on comparisons between primary tumors and their matched metastases in 27 samples from 10 cases sequenced at an average depth of 150 × (Fig. 5a). Additional data from cases analyzed with single-region sequencing are shown in Supplementary Figure S6. By combining genome-wide analysis of private and shared single nucleotide variants (SNVs) and insertions/deletions (Indels) with manually curated variants in known cancer driver genes (Supplementary Data File S2), we generated phylogenetic reconstructions for each patient (Fig. 5). In all cases, this analysis revealed that the last common ancestor accumulated a very large number of mutations, regardless of histotype or smoking history, when compared with private variants in the corresponding primary tumor and its derivative metastases (Fig. 5a). Although single-site sampling of all but two of the primary tumors in our study means that we cannot comprehensively model intratumor heterogeneity, these data suggest that metastatic lesions are not highly divergent from the primary tumor.

In a parallel analysis, we considered the degree of heterogeneity based on shared and private copy number events (Fig. 5b). Consistent with prior observations [10, 11], considerable heterogeneity in CNV was observed, suggesting that ongoing chromosomal instability is important in generating clonal diversity within primary tumors, and between primary tumors and their derivative metastases [2]. However, diversity based on CNV was not universal, with three cases (LUAD3, LUAD6, and SCLC2) exhibiting remarkable similarity between primary and metastatic tumors (Fig. 5b). These data support the idea that genomic heterogeneity in lung cancer is largely driven by CNV, but it may not be a universal mechanism for generating clonal diversity.

### Somatic signatures in private mutations

**Fig. 6:**
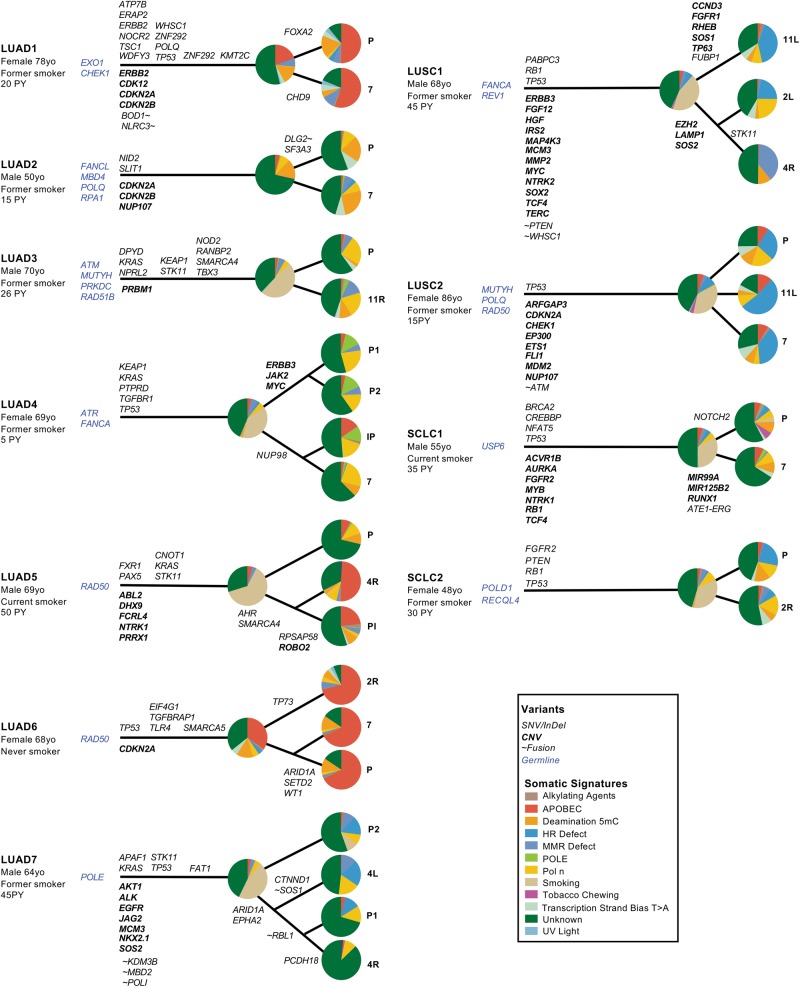
Lung cancer progression models derived from multi-region whole-genome sequencing. Somatic variants by affected genes are shown from left–right according to variant allele frequency. Germline variants are shown immediately to the left of the putative point of origin of each tumor. Somatic signature pie charts depict events in the common ancestor and private to each site. P primary tumor; metastases are indicated by lymph node station except for Pl (pleura) and IP (intrapulmonary). LUAD lung adenocarcinoma, LUSC lung squamous cell carcinoma, SCLC small cell lung cancer

In early-stage lung cancer, mutational diversity following the establishment of the last common ancestor has been associated with a somatic signature consistent with the activation of APOBEC enzymes [10, 11], an endogenous mutagenic process thought to be driven by inflammation [24]. To determine whether this process was seen in metastatic lung cancer, we analyzed somatic signatures in events private to both primary and metastatic tumors from the 11 patients with informative multi-region samples. Although we observed a strong APOBEC signature in private events in three LUAD cases (LUAD1, 2, 6; Fig. 6), private events in the remaining cases exhibited marked diversity in somatic signatures between patients, and between sampling sites within the same patient (Fig. 6). Excluding variants driven by as yet unknown mechanisms, private events in the remaining cases were dominated by patterns consistent with deamination of 5-mC (LUAD2; Fig. 6), Polymerase Eta (Polη) (LUAD3, 4; Fig. 6), or defects in homologous recombination (HR) (LUAD7, LUSC1, 2; Fig. 6). Unexpectedly, we also observed heterogeneity in private somatic signatures between samples within individual patients (Fig. 6), suggesting that the acquisition of SNV/Indels during progression from a common ancestor may also diverge along different paths.

**Fig. 7:**
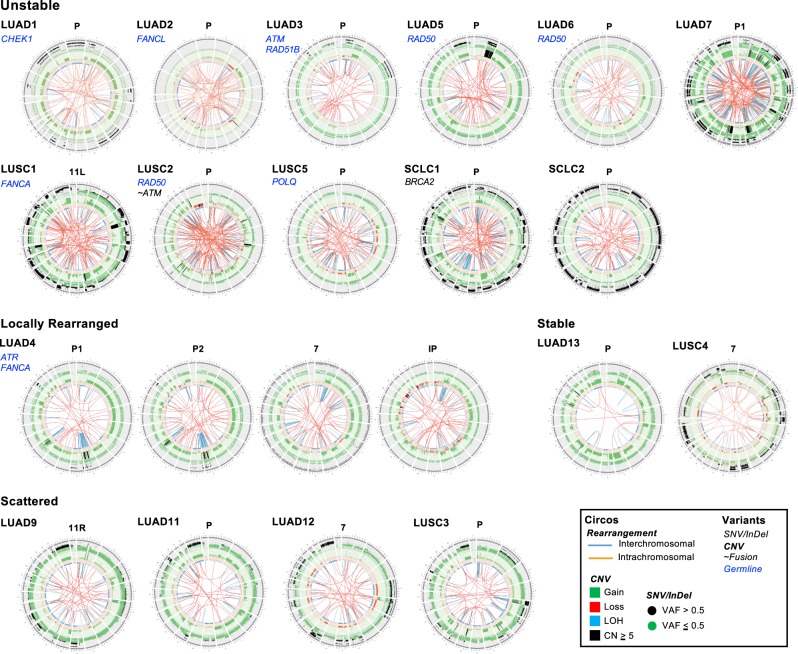
Circos plots from samples analyzed by multi-region and single-region WGS with patterns of structural variants consistent with unstable, locally rearranged, scatted and stable genomic signatures. P primary tumor; metastases are indicated by lymph node station except for Pl (pleura) and IP (intrapulmonary). LUAD lung adenocarcinoma, LUSC lung squamous cell carcinoma, SCLC small cell lung cancer

To explain these results, we first scrutinized our mutation and CNV data to determine if private driver mutations or copy number events in genes relevant to each somatic signature could be identified. In each case, we could not identify a somatic mutation private to one or more sampling regions that might explain the wide spectrum of private somatic signatures we observed. This result raises the possibility that variants established in the common ancestor might influence the pattern of private mutations through interactions with intrinsic or extrinsic factors unique to each subclonal population. To test this idea, we considered truncal somatic events, as well as the germline variants in DNA repair genes identified in our germline analysis as potential mechanisms. In LUAD2, private mutations dominated by a signature for deamination of 5-mC (Fig. 6) were associated with germline variants in two genes functionally implicated in the repair of these mutations, *MDB4* [25] and *RPA1* [26] (Fig. 6; Supplementary Table S3). Private events in two cases of LUSC were dominated by a signature suggestive of a defect in HR DNA repair (Fig. 6) and in both cases, germline variants with somatic LOH were detected in HR DNA repair genes *FANCA* in LUSC1 and *RAD50* in LUSC2. (Fig. 6; Supplementary Table S3; Supplementary Data File S4). Although we could not make such an association in cases with private event signatures dominated by APOBEC (Fig. 6; Supplementary Figure S5) or Polη (Figs. 6, 7), these data suggest that germline variants in a different subset of DNA repair genes may influence the acquisition of private somatic mutations during lung cancer progression, distinct from those associated with mutation burden in the founding clone.

### SVs and chromosomal instability

The extent and complexity of somatic SVs in cancer can be associated with chromosomal instability, an important driver of tumor progression and genomic heterogeneity [27]. Moreover, SV patterns identified by WGS can distinguish tumors with transient periods of instability leading to focal chromosomal rearrangements from those with ongoing, genome-wide instability [13]. Most samples in our cohort demonstrated an SV pattern associated with highly unstable genomes that was shared between primary and metastatic tumors (Fig. 7; Supplementary Figure. S7). In all but three of these cases, we detected potentially deleterious germline variants in at least one DNA repair gene known to participate in the maintenance of chromosomal stability (*CHEK1*, *FANCL*, *ATM*, *RAD51B*, *RAD50*; Fig. 7; Supplementary Table S3). LUAD4 exhibited a distinct focal rearrangement pattern that diverged between primary and metastasis, also associated with germline variants in *ATR* and *FANCA* (Fig. 7). In the primary tumor, a prominent chromothriptic pattern was seen in chromosome 8, whereas a distinct event was seen in chromosome 1 in both metastases (Fig. 7).

With the exception of LUSC2, which harbored a somatic LOF fusion in *ATM* (Figs. 2, 7; Supplementary Table S2), and SCLC1, which contained a somatic event in *BRCA2*, we did not detect shared or private events in genes associated with chromosomal instability. Additional Circos plots, along with an overview of additional genomic data for each case is shown in Supplementary Figure S8. In keeping with our somatic signature analysis, these data suggest that germline variants in DNA repair genes may also play a role in the progression of metastatic lung cancer through the generation of chromosomal instability.

## Discussion

The propensity of lung cancer to spread within the chest prior to diagnosis is a major barrier to the delivery of curative therapy [28]. This long-standing clinical problem is likely to be driven by multiple factors, including the challenges associated with early screening, and the biological virulence of lung cancer. In addition, lung cancers have ready access to an abundant blood supply and complex lymphatic system that can promote metastasis at a very early-stage of tumor development [29]. Using EBUS-TBNA sampling and high-depth WGS, we have demonstrated the genomic complexity and heterogeneity underlying the process of lung cancer metastasis in the chest. As part of this analysis, we detected mutations in tumor-suppressor genes not normally associated with lung cancer, novel LOF fusion events in critical tumor suppressors including *PTEN* and *ATM*, and passenger mutations in non-driver genes such as *ATP7B*, *ERAP2*, and *TLR2* that may have functional and/or therapeutic significance. The degree to which these findings differentiate early from late-stage lung cancer will require a comprehensive genomic evaluation in a large number of patients with metastatic disease.

The large number of variants identified through WGS allowed us to accurately map somatic signatures in our sample set. The degree of variation in the smoking signature of tumors in ever-smokers was surprising given the substantial pack-year exposure in most cases, and the high somatic mutation burden even in cases in which the somatic smoking signature was less dominant. Several potential explanations for this result can be considered. First, it is possible that inflammation caused by smoking, and its associated mutagenic effects, may predominate in some patients [30]. This idea is supported by the dominant APOBEC signature seen in three former-smokers. Second, it is possible that environmental effects other than smoking that drive lung cancer initiation in both former-smokers and never-smokers may be important, and that the interpretation of somatic signatures with respect to carcinogens such as second-hand smoke, radon, indoor air pollution, and occupational exposures [31] may need to be evaluated. Third, it is possible that germline variants contribute to the effect of smoking on the pattern of lung cancer mutations. Our data strongly suggest that germline variants in genes implicated in the repair of smoking-induced DNA damage may contribute to the somatic signatures observed in tumors from current- and former-smokers. If this assertion can be substantiated in larger prospective studies, it would suggest that such variants may only be penetrant in the setting of cigarette exposure, and may contribute to lung cancer risk only in smokers.

Several studies have strongly implicated APOBEC activation as a driver of mutagenesis in lung cancer progression [10, 11], although this seems to be less prevalent in EGFR mutant LUAD in Asians [32]. With respect to mutations associated with metastatic lung cancer progression, our data suggest that this process is heterogeneous both within and between patients, with private somatic signatures of HR deficiency or deamination of 5-mC present in addition to APOBEC signatures. In three cases, we identified germline variants that could be functionally associated with these signatures. Similarly, we observed a striking association between highly rearranged somatic genomes and germline variants in genes such as *ATM*, *ATR*, *FANCA*, and *RAD51B*. These results suggest that extrinsic factors unique to the environment of each metastasis may interact with germline variants to influence the mutation spectrum in metastatic disease. Large-scale prospective studies are needed to further validate these results in patients with distant metastatic disease. Given the connection between tumor mutation burden and the immune response [33], our data suggest that clinical trials in which the genomic heterogeneity in metastatic lung cancer in compared with the response to immunotherapy would be highly informative.

Given the poor prognosis of metastatic lung cancer, insights into the genomic evolution of this disease are urgently needed. In a recently published study, Um and colleagues used EBUS-TBNA sampling to perform multi-region exon capture and RNAseq analysis on six cases of advanced lung cancer [34]. In keeping with our results, mutations in critical genes, such as *TP53* and *RB1*, were shared across all sites in individual patients. They also observed variability in the degree of mutational heterogeneity, suggesting distinct models of clonal evolution in which metastatic spread occurs early or late during tumor progression. Although our results are consistent with previous observations that primary lung cancers are highly heterogeneous [10, 11], we find that mutational heterogeneity in metastatic disease is far more limited with respect to both driver and passenger genes, in keeping with a recent WGS study in four cases of metastatic pancreatic cancer [16]. Also in keeping with previous reports of multi-region sequencing in early-stage lung cancer, we found that metastatic heterogeneity is largely driven by CNV [10, 11].

Our data are consistent with two models of tumor evolution in metastatic lung cancer. In the first model, a slowly growing primary tumor progressively acquires a very large number of SNVs and Indels, followed much later by the establishment of metastases. However, such a model seems at odds with the clinical and biological behavior of lung cancer. Alternatively, our results could be explained by prolonged exposure of the airway to carcinogens, followed by rapid progression associated with chromosomal instability, accelerated growth, and metastasis. The airway epithelium is a low turnover cell population continuously exposed to environmental toxins, bacteria, viruses, and oxidative stress [35]. In addition, oncogenic mutations have been observed in the normal airway epithelium of ever-smokers [36, 37], suggesting that mutant airway epithelial clones can persist for decades. Given the lack of ready access to well-defined precursor lesions in lung cancer research [38], detailed genomic analysis of normal airway epithelium in ever-smokers with lung cancer may provide critical insights into the early events driving the establishment and progression of lung cancer. More detailed prospective studies will be needed to address whether the histologic subtypes of lung cancer, particularly adenocarcinoma, relate to environmental exposure, smoking, and germline variants. Finally, our results suggest that gene-by-environment interactions in the DNA repair pathway may influence the mutational pathogenesis of lung cancer during tumor initiation, progression and metastasis.

## Materials and methods

### Subject cohort and sample processing

Patients undergoing EBUS-TBNA for diagnosis and/or staging gave prospective written informed consent to participate in this study. The study was approved by the St Vincent’s Hospital human research ethics committee protocol number SVH14–256. Once the on-site cytologist confirmed the diagnostic material had been obtained for clinical purposes, an extra sample was taken for research purposes, suspended in sterile saline, and placed on ice.

In the laboratory, each sample was disaggregated, centrifuged, and re-suspended in 450 μl of PBS. Next, a 50 μl aliquot was removed to generate a cytology smear to determine tumor cellularity of the research specimen. The cytology specimen was stained with DiffQuik, and then reviewed by a cytopathologist to estimate the fraction of the sample made up of tumor cells, as well as sample quality. Samples containing > 20% tumor cells were then processed for DNA extraction from the frozen cell suspension using the Qiagen (Hilden, Germany) DNEasy kit as per the manufacturer’s instructions, and then checked for quality, purity, and integrity in preparation for WGS. Germline DNA was obtained using the same methodology from peripheral blood mononuclear cells.

### WGS

Sequencing was carried out on the Illumina (San Diego, CA, USA) HiSeq X Ten platform with a paired-end read length of 150 bases. We sequenced all germline (peripheral blood buffy coat) samples to a minimum mean coverage of 30 × and all tumor samples to a minimum mean coverage of 60 × . In cases where multi-region samples were available, samples with an inferred cellularity of >15% were sequenced to an additional minimum mean coverage of 90 × , giving a total of at least 150 × .

### Bioinformatics

The analysis pipeline was conducted using the Seave platform [39]. Somatic SNVs and Indels were called using Strelka v2.0.17.strelka1 [40] and the GATK Best Practices workflow [41]. Final variants were annotated for their impact on the genome using Variant Effect Predictor v87 [42]. Variants were filtered using a CADD [42] scaled score of >2 (if available) and a maximum allele frequency of 1% for somatic variants and 2% for germline variants in each of ExAC [43]. Somatic variant filtration was performed using two parallel approaches. First, we generated a consensus cancer gene driver list that includes pan-cancer driver genes [44], and putative lung cancer driver genes [7–9] (Supplementary Data File S1). Candidates were then identified by filtering on Strelka QSS/QSI > 15, impact severity, and PolyPhen/PROVEAN/SIFT prediction, and then annotated using PubMed, IntOGen [45] and Varsome (www.varsome.com). In the second approach, we queried all genes and identified additional LOF candidates by filtering using the same filtration approach with the added criteria of evidence for somatic LOH. Candidates were then manually annotated by searching for evidence of a potential link between cancer pathogenesis or therapeutic responses using PubMed.

Germline variants were analyzed using a consensus list of DNA repair genes generated by searching the Gene Ontology database [46] (Supplementary Data File S2). Missense variants were further filtered according to the following criteria: (ClinVar annotation pathogenic or likely pathogenic) OR (variant in a Pfam domain AND defined as damaging by PROVEAN OR SIFT or PolyPhen) OR (defined as damaging in at least two of either PROVEAN, SIFT or PolyPhen). LOH events were confirmed by comparing the variant allele frequency (VAF) in germline and tumor samples with inferred tumor cellularity. Somatic signatures were analyzed using the SomaticSignatures R package (v2.6.0) [47]. Somatic signatures for TCGA LUAD and LUSC data were determined using somatic variants called by MuTect2 and obtained from TCGA. Variant call files (VCFs) were subset to autosomal chromosomes and PASS variants were used only, all other analysis was identical to the EBUS-TBNA data.

Somatic CNVs were identified using Sequenza v2.1.0 [48] using a bin size of 200 and a minimum total depth of 20. Candidate CNV events were identified by filtering for estimated copy number 0 or > 6 and segment size > 20 kb. Events were then verified by identifying a corresponding event using Manta v0.27.1 [49], and manually with IGV. Candidates were then annotated by searching the PubMed, COSMIC [50], and CBioPortal [51]. SV calling and fusion analysis was performed with Manta v0.27.1 [49] and Oncofuse v1.0.9b2 [52]. Further details of describing the bioinformatic analysis are presented in Supplementary Information. Sequencing data are available at the European Nucleotide Archive (https://www.ebi.ac.uk/ena) under accession number PRJEB28616.

## Electronic supplementary material

Supplementary Information

Supplementary Data File S1

Supplementary Data File S2

Supplementary Data File S3

Supplementary Data File S4

Supplementary Data File S5
