# Supplementary material for: Deep multi-region whole-genome sequencing reveals heterogeneity and gene-by-environment interactions in treatment-naive, metastatic lung cancer
Source: Oncogene. 2018 Oct 22;38(10):1661–75. doi: 10.1038/s41388-018-0536-1 (PMC6462862; doi:10.1038/s41388-018-0536-1)
Supplement: Supplementary file 1 — Supplementary Information [file 41388_2018_536_MOESM1_ESM.pdf]

SUPPLEMENTARY FIGURES

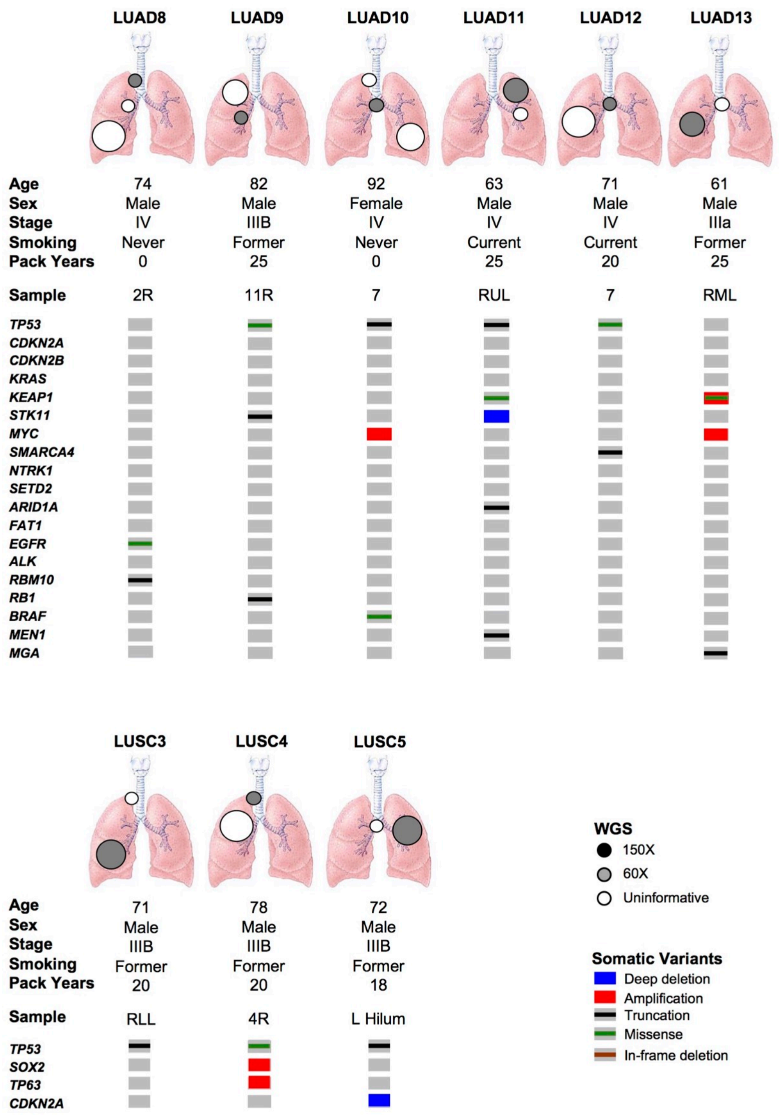

Figure S1. Overview of cases analyzed with single-region whole genome sequencing. Oncoprint outputs depicting SNV, Indel and CNV events in lung cancer driver genes for each sample are shown with heterogeneous mutations highlighted. LUAD = lung adenocarcinoma; LUSC = lung squamous cell carcinoma.

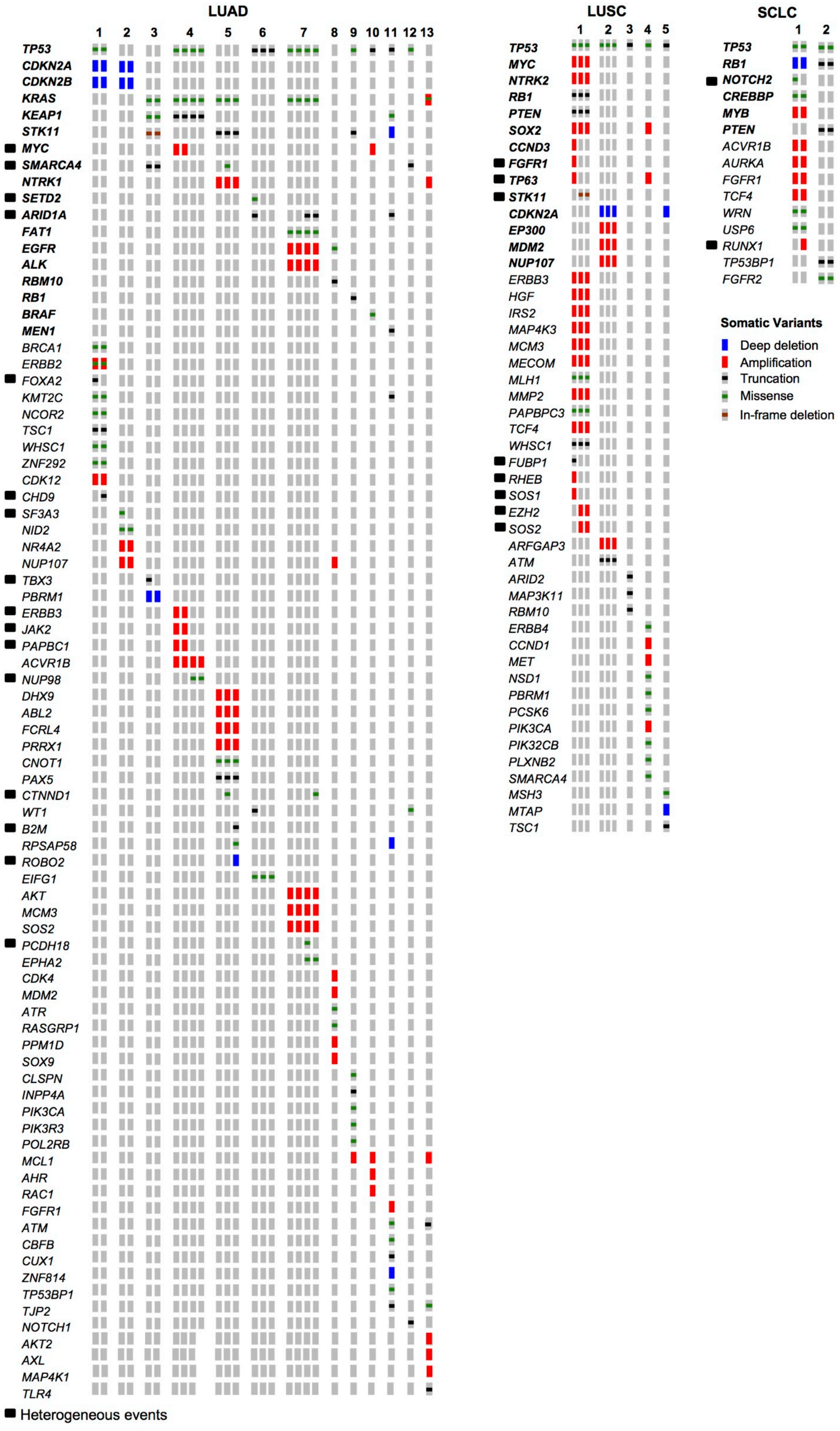

Figure S2. Overview of all cases analyzed with whole genome sequencing. Oncoprint outputs depicting SNV, Indel and CNV events in pan-cancer driver genes for each sample are shown with heterogeneous mutations highlighted. Lung cancer driver genes are highlighted in bold. LUAD, lung adenocarcinoma; LUSC, lung squamous cell carcinoma; SCLC, small cell lung cancer.

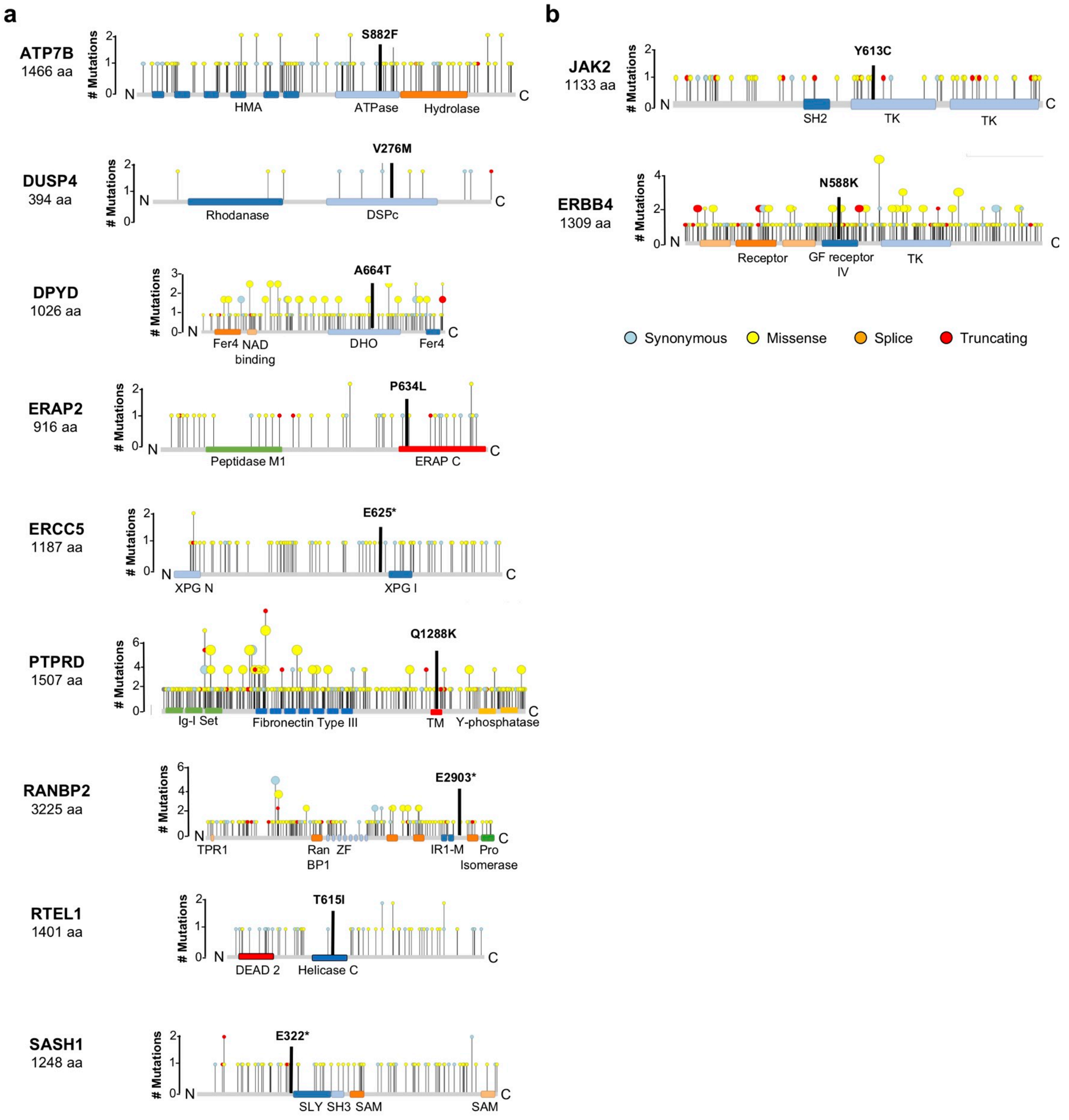

Figure S3. Somatic mutations in non-driver genes of potential significance. (**a**) Candidate loss-of-function variants associated with loss-of-heterozygosity. (**b**)**,** Candidate gain-of-function mutations. Needle plots generated by IntOGen depict somatic mutations in cancer described for each gene product. Protein domains are indicated using Pfam nomenclature.

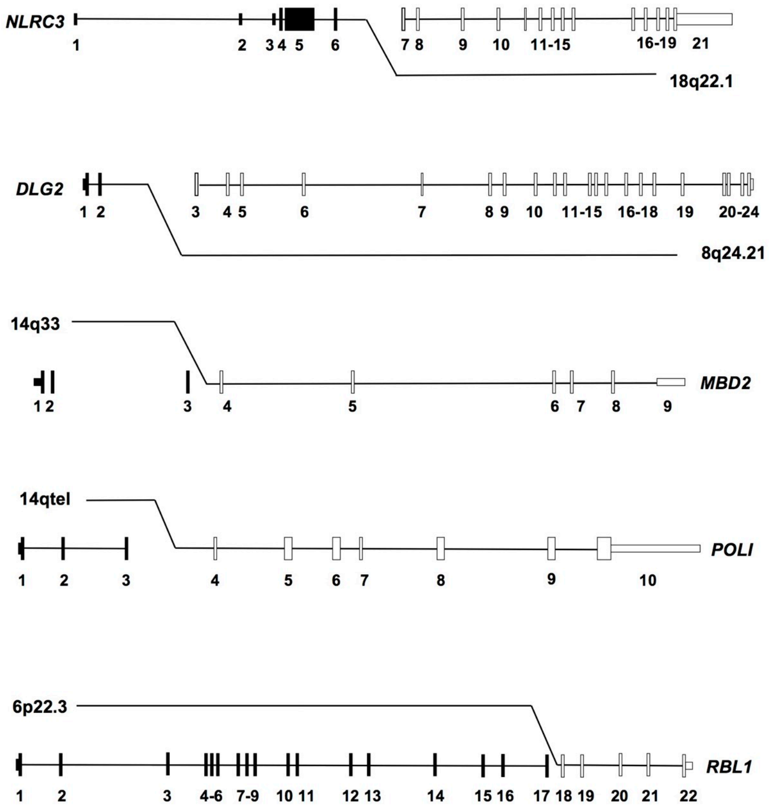

Figure S4. Examples of candidate loss-of-function fusions predicting truncation events in non-driver genes of potential significance. Exons in each fusion partner are numbered.

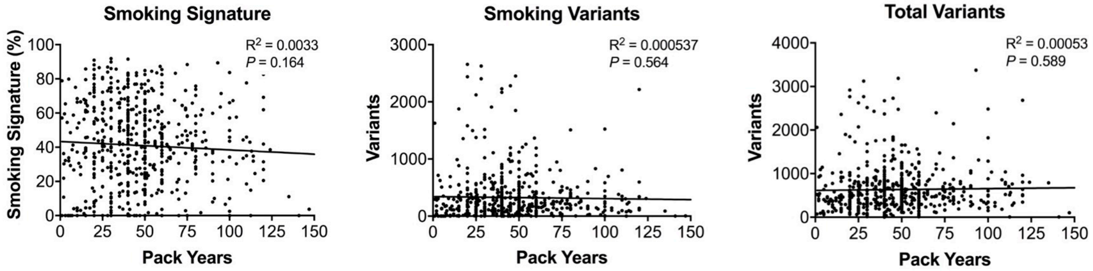

Figure S5. Linear regression analysis comparing pack year cigarette exposure with somatic smoking signature, the number of smoking-related variants and total somatic variants in the TCGA lung cancer data set. n = 581.

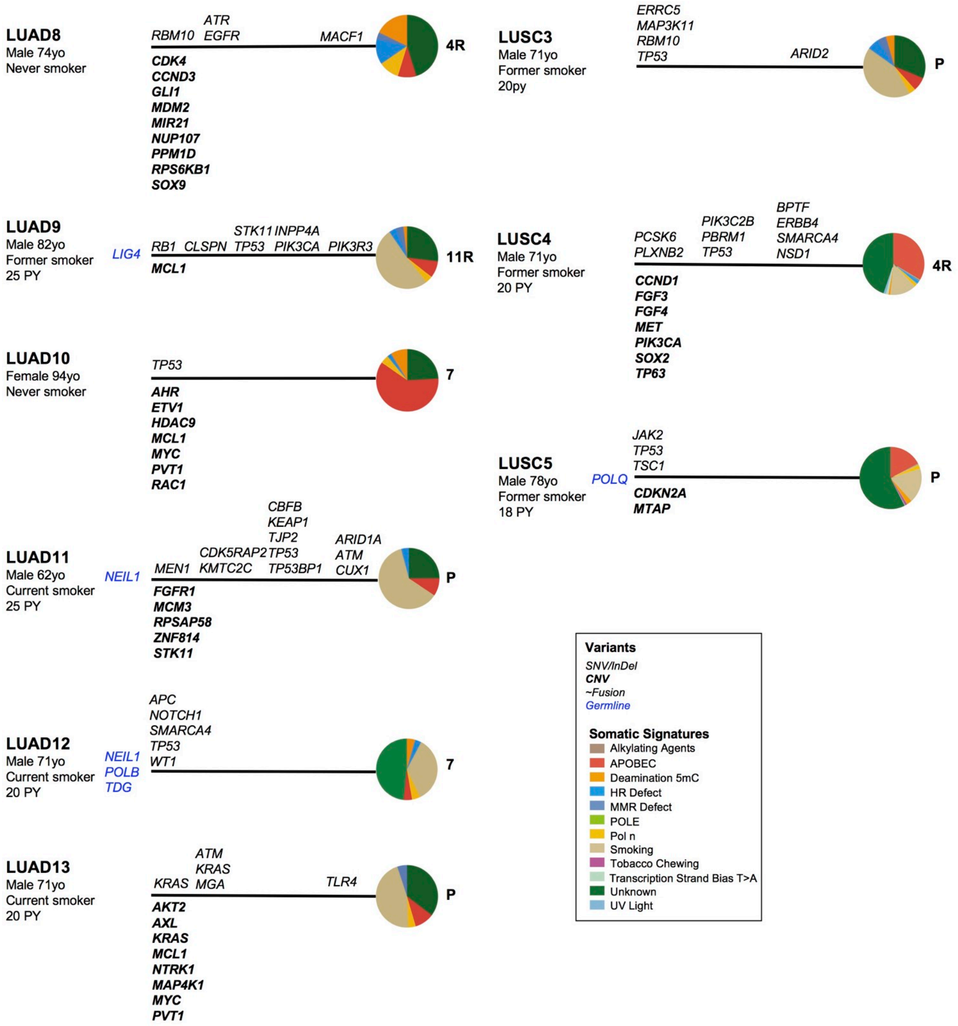

Figure S6. Additional cases of metastatic lung cancer analyzed by single-region whole genome sequencing. Somatic variants are shown from left-right according to variant allele frequency. Germline variants are shown immediately to the left of the putative point of origin of each tumor. LUAD = lung adenocarcinoma; LUSC = lung squamous cell carcinoma; SCLC = small cell lung cancer.

Figure S7. Circos plots from multi-region samples analyzed by WGS with patterns of structural variants consistent with unstable genomes. P, primary tumor; metastases are indicated by lymph node station except for Pl (pleura) and IP (intrapulmonary). LUAD, lung adenocarcinoma; LUSC, lung squamous cell carcinoma; SCLC, small cell lung cancer.

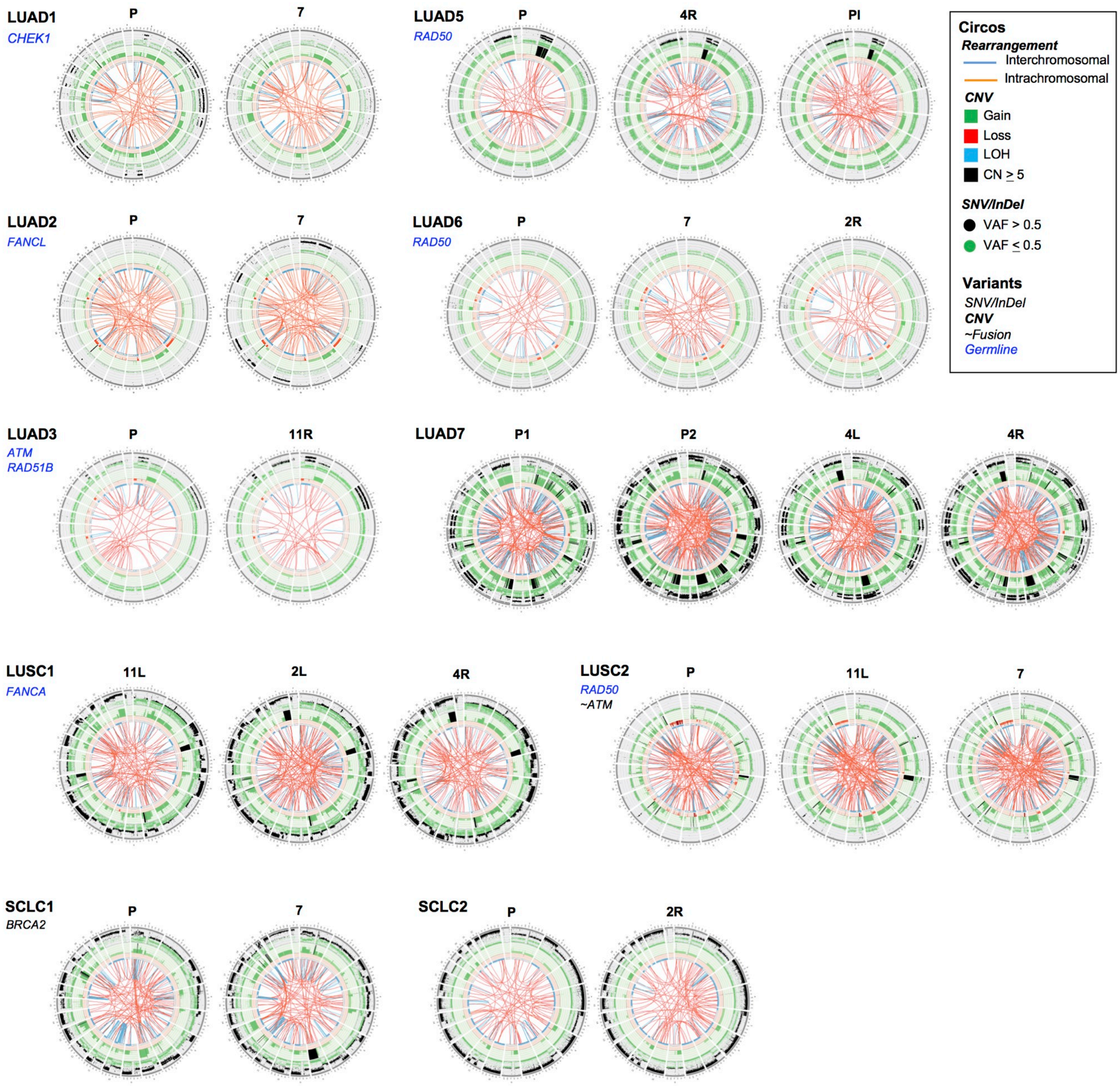

Figure S8. Overview of data summarized case-by-case.

LUAD1

Stage IIIA
Female 78yo
Former smoker 20PY
Primary: LLL
Metastasis: LN 7

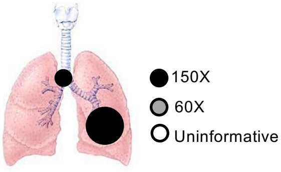

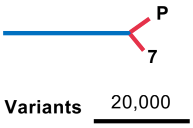

IntoGen drivers in ***Bold***
Het in germline

Private to Primary

FOXA2	H348fs

Shared

ATP7B	S882F
BOD1	LOF fusion
ERAP2	P365L
ERBB2	L124S
ERBB2	CN Gain
EXO1	G274R
CDK12	CN Gain
CDKN2A	Del
CDKN2B	Del
CHEK1	R36*
KMT2C	D244H
NCOR2	S1971F
NLRC3	LOF fusion
POLQ	E2016A
TP53	
M246I	
TSC1	Q328*
WDFY3	L1058P
WHSC1	E497K
ZNF292	P1530L

Private to LN7

CHD9	T670fs

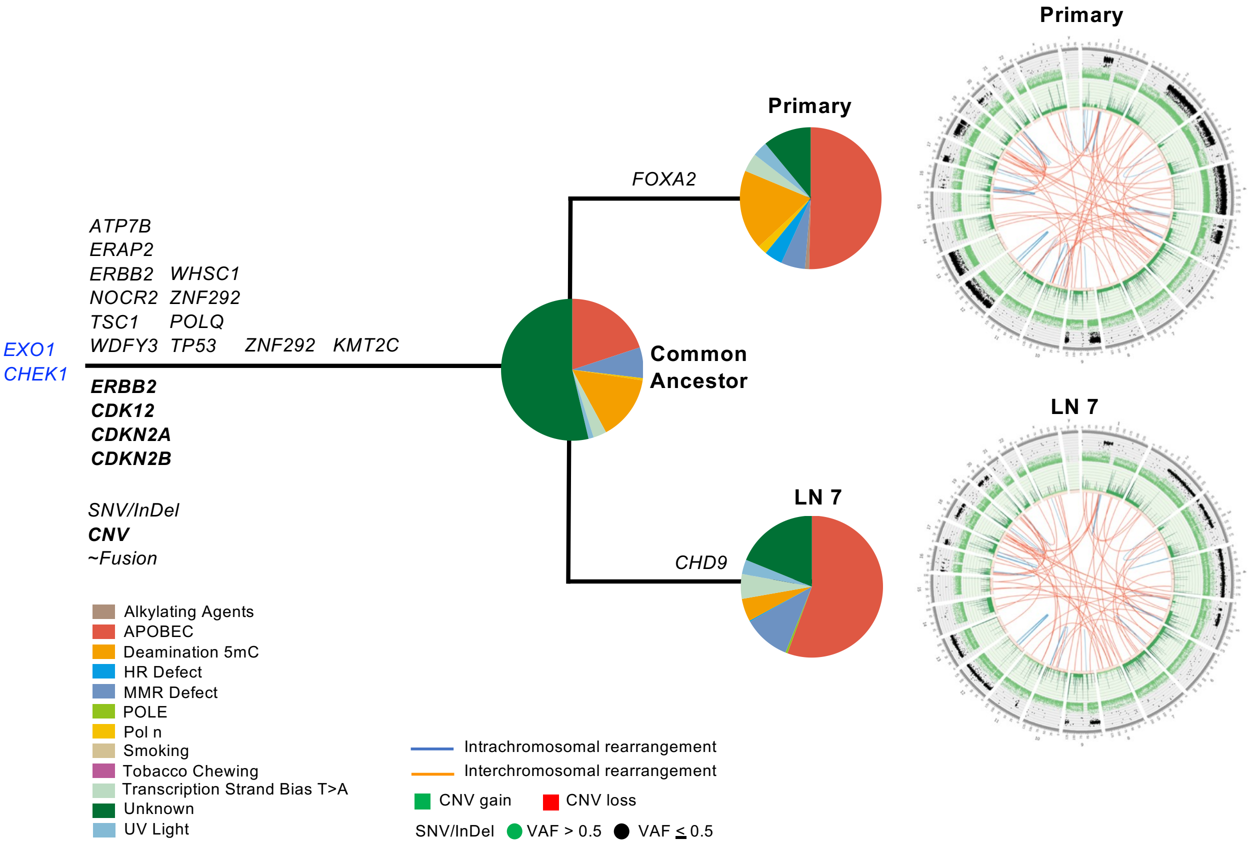

LUAD2

Stage IIIB
Male 50yo
Former smoker 15PY
Primary: RLL
Metastasis: LN 7

IntoGen drivers in ***Bold***
Het in germline

Private to Primary

DLG2	LOF fusion
SF3A3	Y479C

Shared

CDKN2A	Del
CDKN2B	Del
FANCL	P17R
NID2	G855A
NUP107	CN Gain
MBD4	N467S
POLQ	I1420fs
RPA1	R389W
SLIT1	G1146R

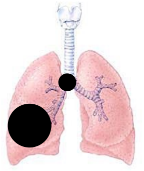

- 150X
- 60X
- Uninformative

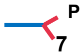

Variants 20,000

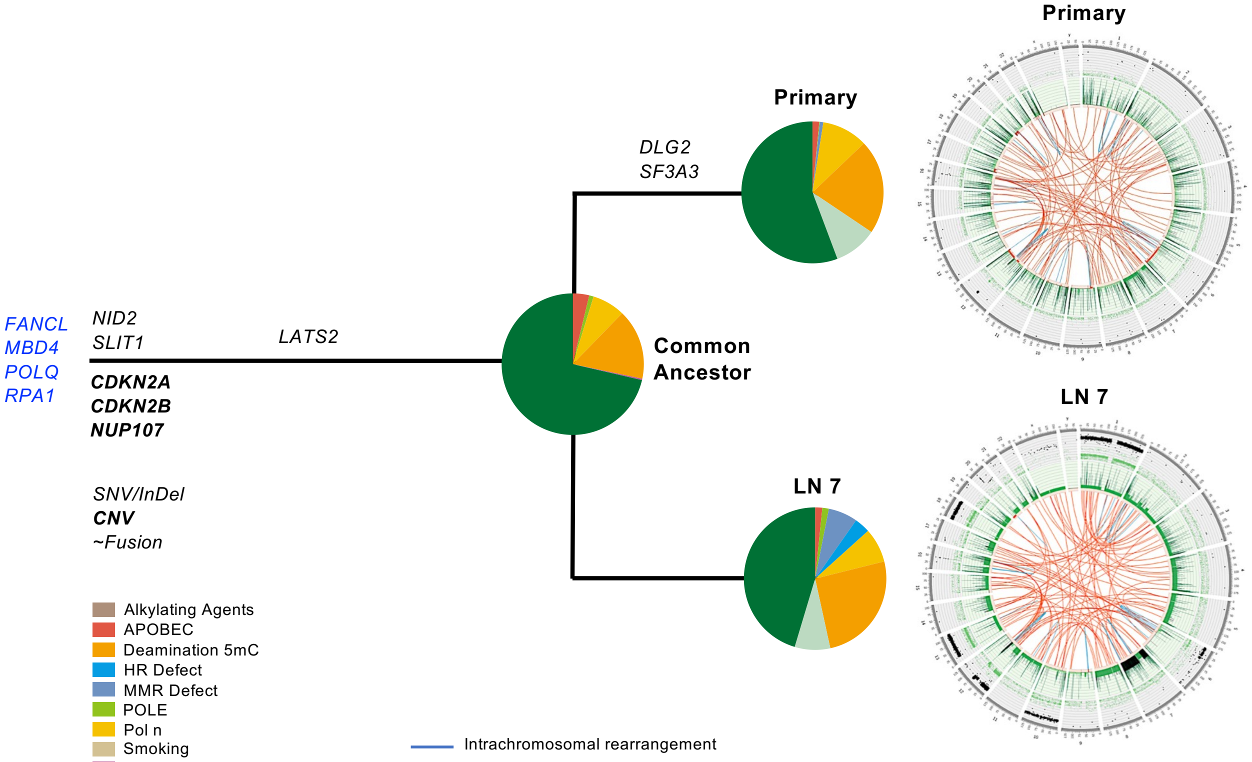

SNV/InDel
CNV
~Fusion

- Alkylating Agents
- APOBEC
- Deamination 5mC
- HR Defect
- MMR Defect
- POLE
- Pol n
- Smoking
- Tobacco Chewing
- Transcription Strand Bias T>A
- Unknown
- UV Light

- Intrachromosomal rearrangement
- Interchromosomal rearrangement
- CNV gain
- CNV loss
- SNV/InDel: VAF > 0.5, VAF ≤ 0.5

LUAD3

Stage IIIA
Male 70yo
Former smoker 26PY
Primary: RUL
Metastasis: LN 11R

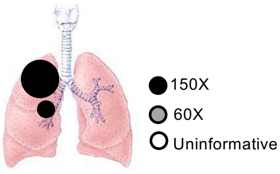

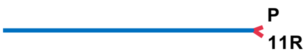

Variants 20,000

IntoGen drivers in ***Bold***
Het in germline

Shared

DPYD	A664T
KEAP1	G417V
KRAS	G12C
NOD2	L214fs
NPRL2	K104R
MUTYH	V207M
PBRM1	HozDel
PRKDC	L1706Q
RANBP2	E2903*
SMARCA4	Splice site
STK11	IFD
TBX3	A494fs

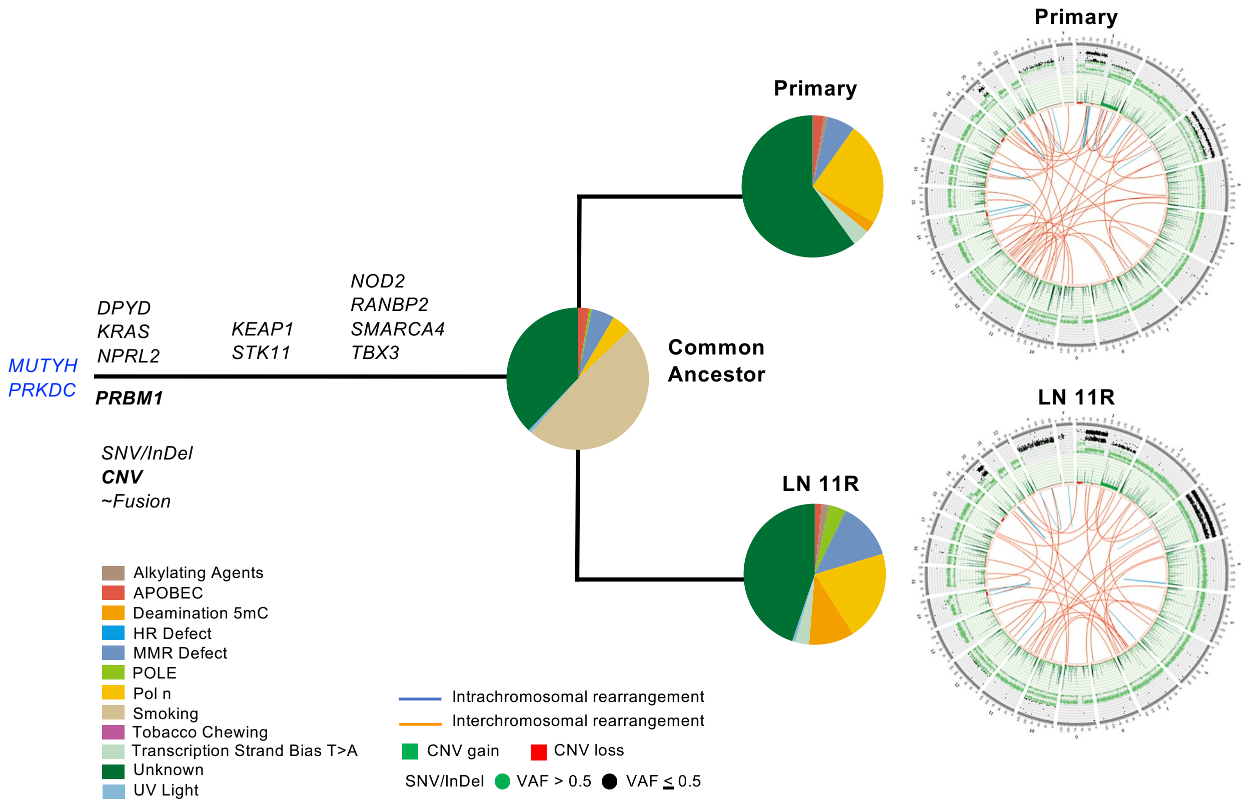

LUAD4
Stage IV
Female 69yo
Former smoker 5PY
Primary: RUL Inner P1; Outer P2
Metastasis: LN 7
Metastasis: RLL Intrapulmonary

IntoGen drivers in ***Bold***
Het in germline

Private to Primary 1,2		Shared		Private to Mets	
ERRB3	CN gain	***ACVR1B***	CN Gain	***NUP98***	L548S
JAK2	CN gain	*FANCD2*	R735Q		
MYC	CN gain	***KEAP1***	Y396*		
PAPBC1	CN gain	***KRAS***	G12D		
		PTPRD	Q1228K		
		TGFBR1	L211V		
		TP53	G245C		

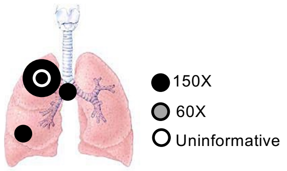

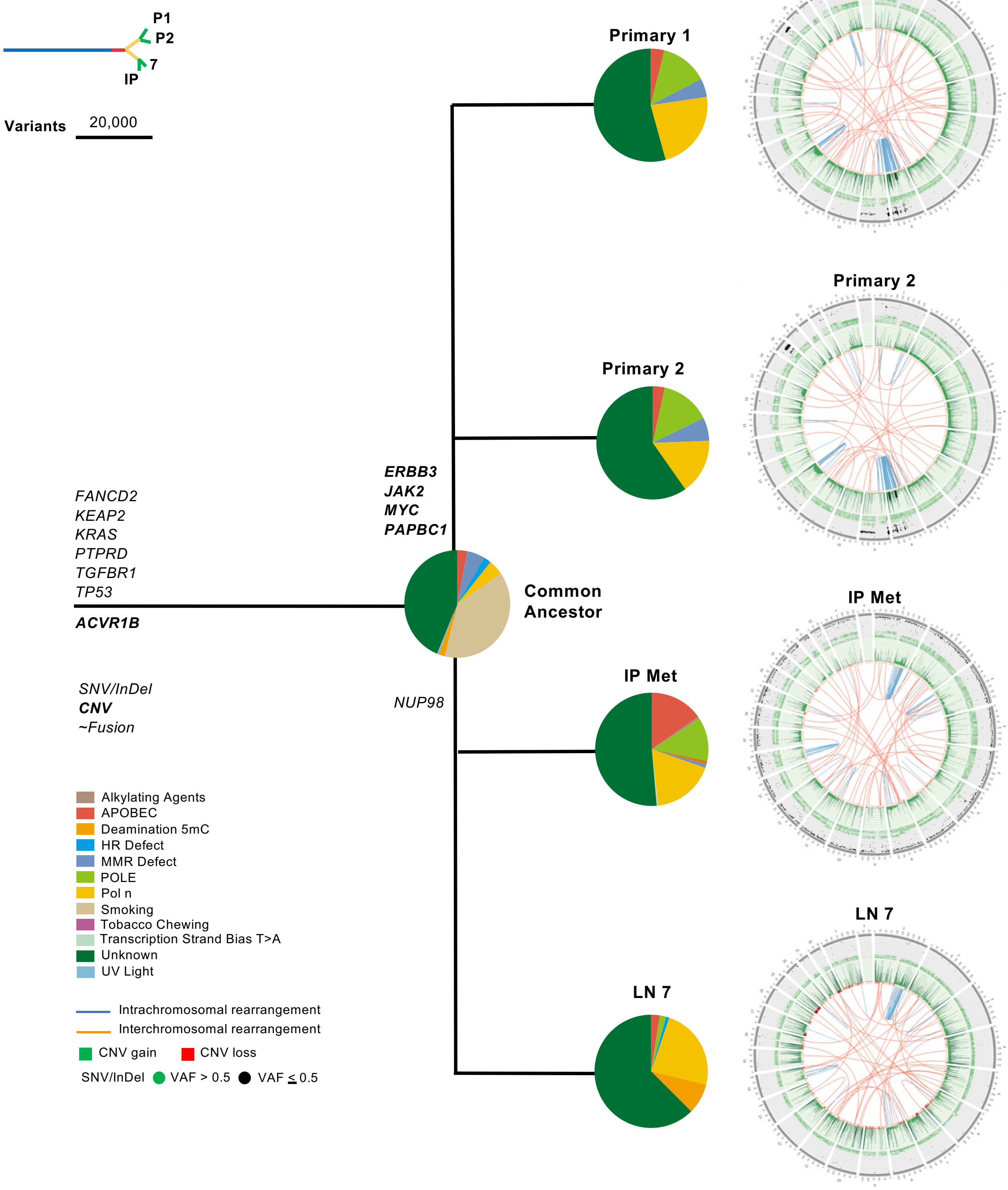

LUAD5

Stage IV
Male 69yo
Current smoker 50PY
Primary: RUL
Metastasis: LN 4R
Metastasis: R Pleura

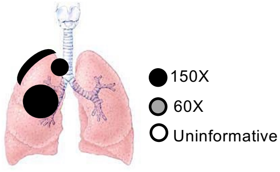

IntoGen drivers in ***Bold***
Het in germline

Shared

ABL2	CN gain
CNOT1	R592L
DHX9	CN gain
FCRL4	CN gain
FXR1	G164V
KRAS	G12C
LIG4	A842D
NTRK1	CN gain
PAX5	S177*
PRRX1	CN gain
RAD50	R1279C

Private to LN 4R

CTNND1	H276Y
SMARCA4	N68H

Private to Pleural Met

ROBO2	HozDel

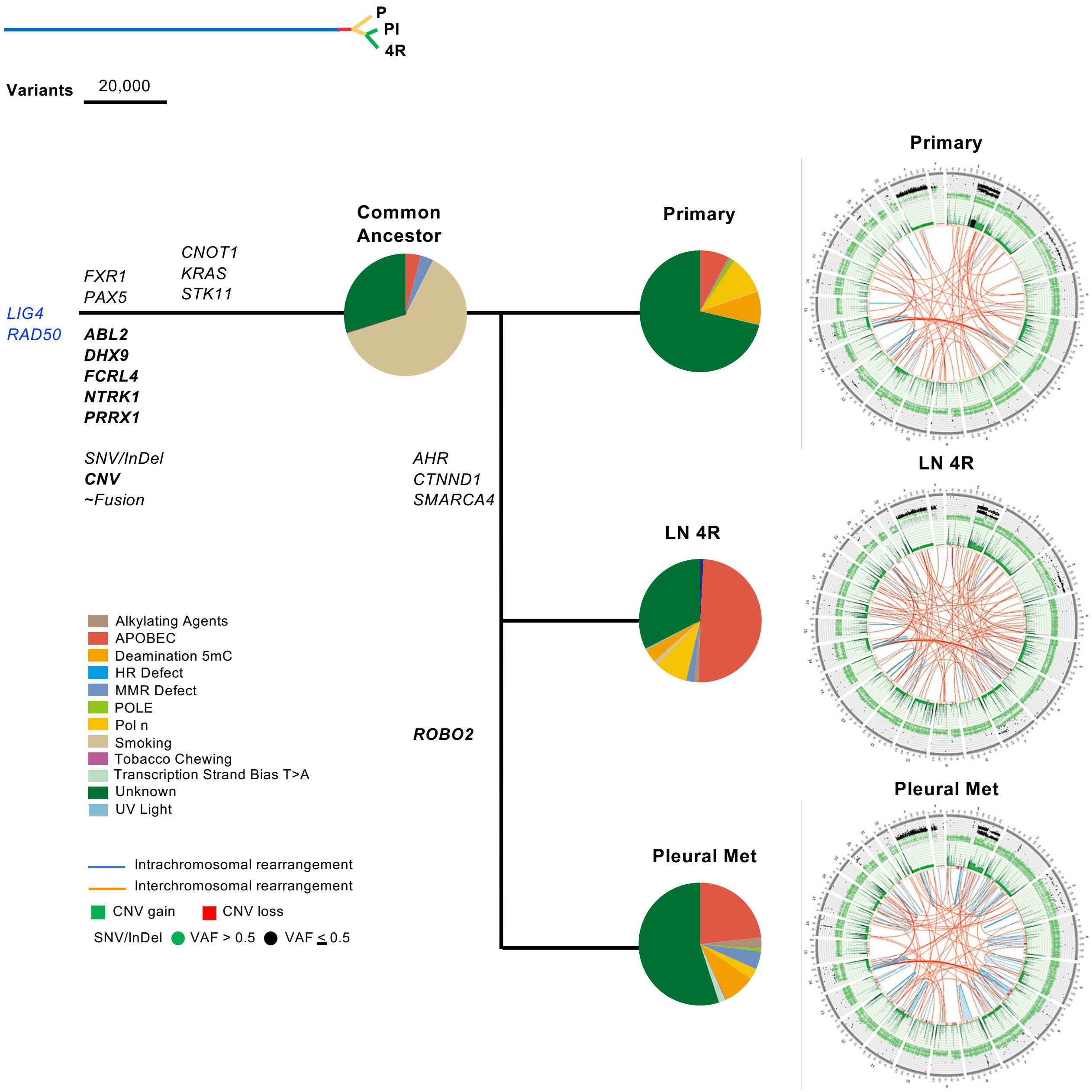

LUAD6

Adenoca Stage IV
Female 68yo
Never smoker
Primary: R Hilum
Metastasis: LN 7
Metastasis: LN 2R

*IntoGen drivers in **Bold***
Het in germline

Private to Primary

ARID1A	Q1493*
SETD2	A1290V
WT1	R462fs

Shared

CDKN2A	HozDel
DROSHA	H983Y
EIF4G1	E792K
RAD50	R884H
SMARCA5	R764*
TGFBRAP1	S809*
TLR4	P568T
TP53	Q104*

Private to Primary + LN7

TP73	T240S

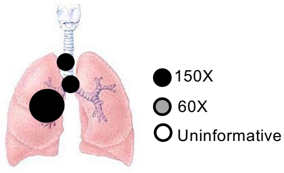

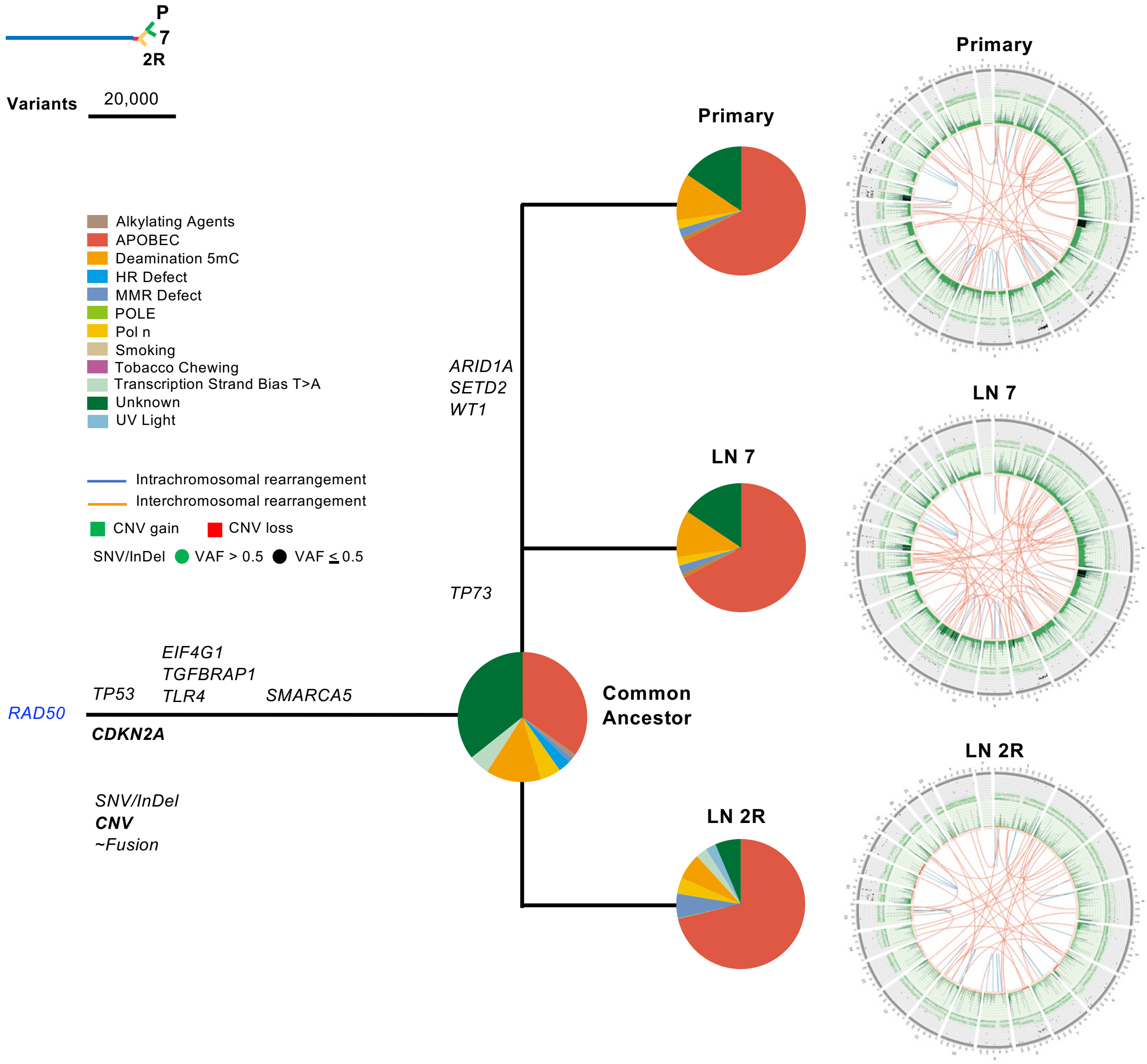

LUAD7

Stage IIIa
Male 64yo
Former smoker 45PY
Primary (P1): RLL
Primary (P2): RLL
Metastasis: (4R)
Metastasis: (4L)

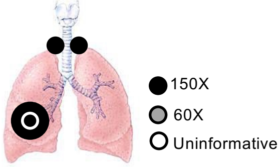

IntoGen drivers in ***Bold***
Het in germline

Private to P1, 4L, 4R

ARID1A	I1176fs
EPHA2	K702R

Private to 4L

*DHX57~**SOS1***	GOF fusion

Private to P1, 4R

RBL1	LOF fusion

Shared

APAF1	Splice
AKT1	CN gain
ALK	CN gain
EGFR	CN gain
FAT1	G4110C
JAG2	CN gain
KDM3B	LOF Fusion
MBD2	LOF Fusion
MCM3	CN gain
NKX2.1	CN gain
STK11	G281fs
KRAS	G12D
POLI	LOF Fusion
POLE	I1127F
SOS2	CN gain
TP53	C141W

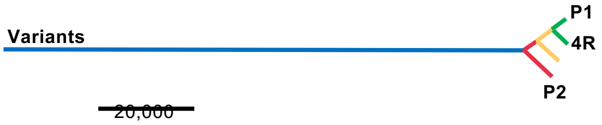

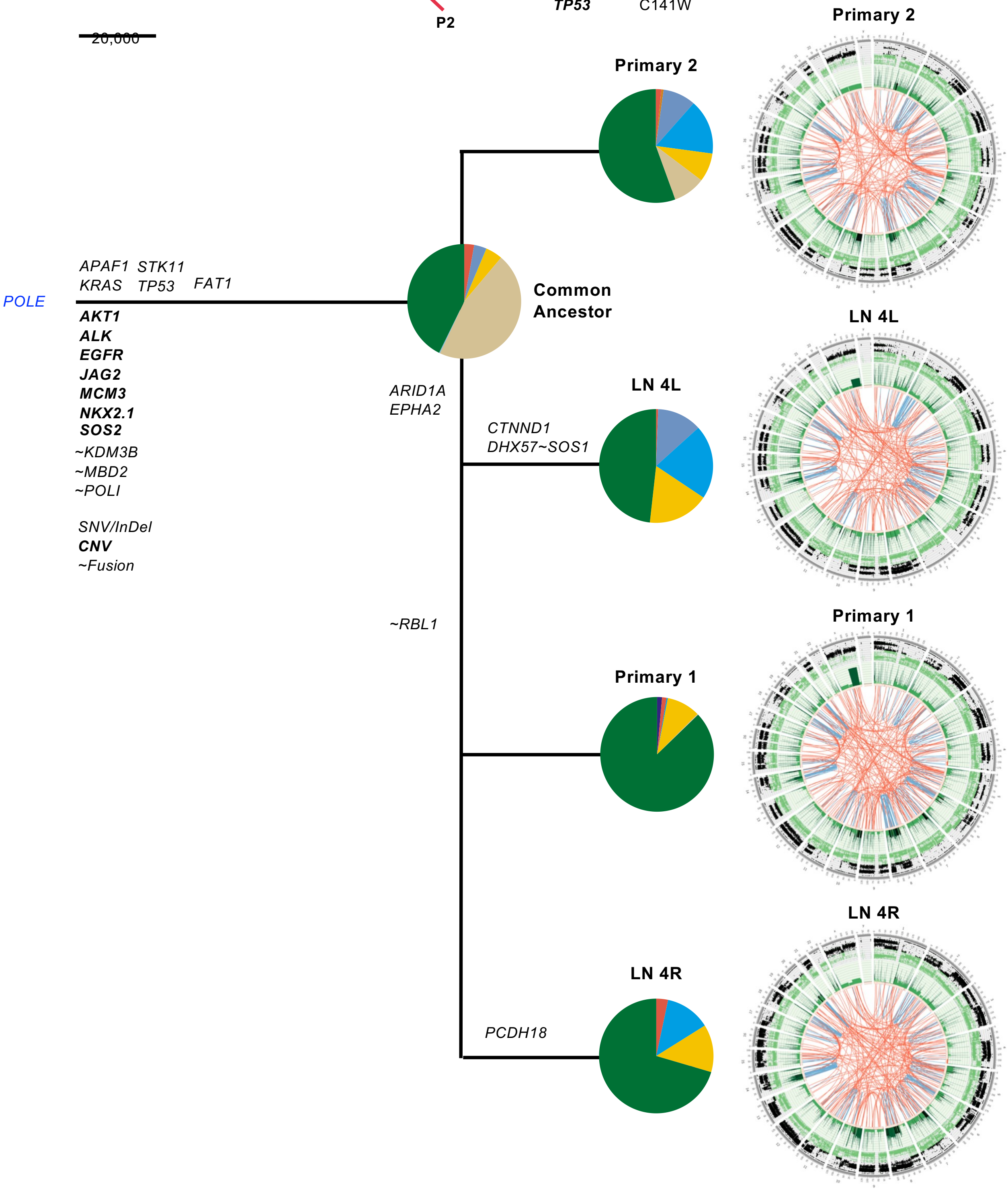

LUAD8

Stage IV
Male 74yo
Never smoker
Primary: Not sampled
Metastasis: LN 11R
Metastasis: LN 4R

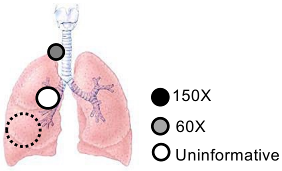

IntoGen drivers in ***Bold***
Het in germline

LN 4R

ATR	Y245N
CDK4	CN Gain
CCND3	CN Gain
EGFR	L858R
GLI1	CN Gain
MDM2	CN Gain
MIR21	CN Gain
NUP107	CN Gain
RASGRP1	D512N
RBM10	L549fs
RPS6KB1	CN Gain
SOX9	CN Gain

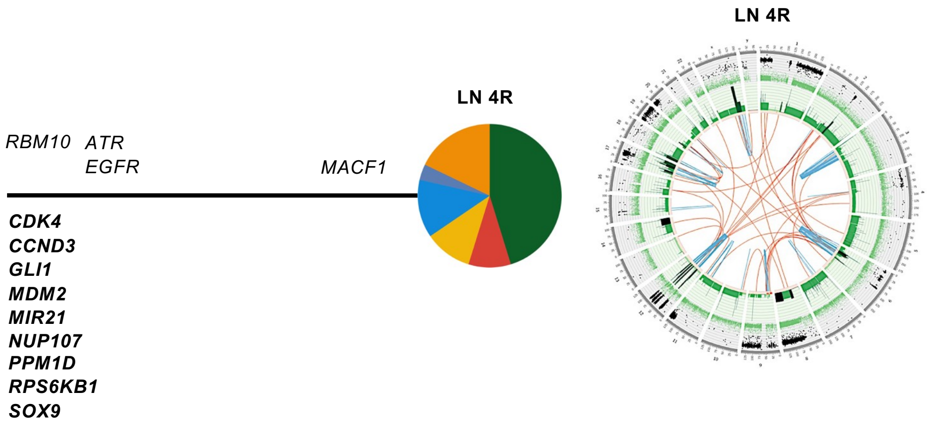

SNV/InDel
CNV
~Fusion

Alkylating Agents
APOBEC
Deamination 5mC
HR Defect
MMR Defect
POLE
Pol n
Smoking
Tobacco Chewing
Transcription Strand Bias T>A
Unknown

Intrachromosomal rearrangement
Interchromosomal rearrangement
CNV gain CNV loss
SNV/InDel VAF > 0.5 VAF ≤ 0.5

LUAD9

Stage IIIB
Male 82yo
Former smoker
Primary: RUL
Metastasis: LN 11R

IntoGen drivers in ***Bold***
Het in germline

LN 11R

CLSPN	S869R
LIG4	A842D
MCL1	CN Gain
PIK3CA	E545K
RB1	Splice
STK11	E199*
TP53	R283P

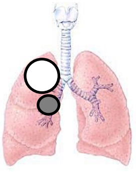

150X
60X
Uninformative

STK11 INPP4A
RB1 CLSPN TP53 PIK3CA
LIG4
MCL1

SNV/InDel
CNV
~Fusion

LN 11R

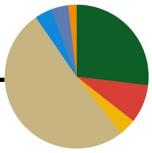

LN 11R

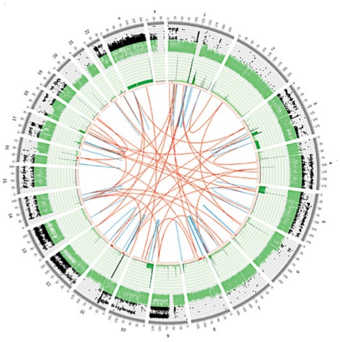

Alkylating Agents
APOBEC
Deamination 5mC
HR Defect
MMR Defect
POLE
Pol n
Smoking
Tobacco Chewing
Transcription Strand Bias T>A
Unknown

Intrachromosomal rearrangement
Interchromosomal rearrangement
CNV gain CNV loss
SNV/InDel VAF > 0.5 VAF ≤ 0.5

LUAD10

Stage IV
Female 92yo
Never smoker
Primary: LLL
Metastasis: LN 7
Metastasis: LN 4R

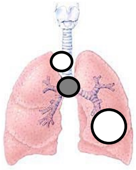

150X
60X
Uninformative

*IntoGen drivers in **Bold***
Het in germline

LN 7

AHR	CN Gain
ETV1	CN Gain
HDAC9	CN Gain
MCL1	CN Gain
MYC	CN Gain
PVT1	CN Gain
RAC1	CN Gain
TP53	P301R
TP53	Q331*

LN 7

TP53

AHR
ETV1
HDAC9
MCL1
MYC
PVT1
RAC1

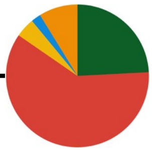

LN 7

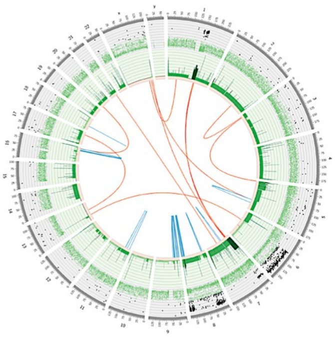

SNV/InDel
CNV
~Fusion

Alkylating Agents
APOBEC
Deamination 5mC
HR Defect
MMR Defect
POLE
Pol n
Smoking
Tobacco Chewing
Transcription Strand Bias T>A
Unknown

Intrachromosomal rearrangement
Interchromosomal rearrangement
CNV gain CNV loss
SNV/InDel VAF > 0.5 VAF ≤ 0.5

LUAD11

Stage IV
Male 62yo
Current smoker 25PY
Primary: LUL
Metastasis: LN 10L

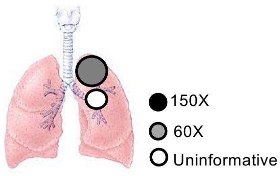

IntoGen drivers in ***Bold***
Het in germline

Primary

ARHGAP35	Del
ARID1A	E1799*
ATM	R2832H
CDK5RAP2	L1455F
CBFB	V174I
FGFR1	CN Gain
KEAP1	R149W
MCM3	CN Gain
MEN1	E547*
NEIL1	Splice
RPSAP58	HomDel
STK11	LOF fusion
TJP2	E14*
TP53	G244fs
TP53BP1	A637S
ZNF814	Del

NEIL1

MEN1 CDK5RAP2

CBFB
KEAP1
TJP2
TP53
TP53BP1

ARID1A
ATM
CUX1

FGFR1
MCM3
RPSAP58
ZNF814
~STK11

SNV/InDel
CNV
~Fusion

Primary

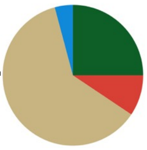

Primary

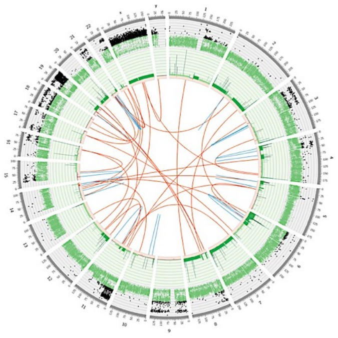

Alkylating Agents
APOBEC
Deamination 5mC
HR Defect
MMR Defect
POLE
Pol n
Smoking
Tobacco Chewing
Transcription Strand Bias T>A
Unknown

Intrachromosomal rearrangement
Interchromosomal rearrangement
CNV gain   CNV loss
SNV/InDel   VAF > 0.5   VAF ≤ 0.5

LUAD12

Stage IV
Male 71yo
Current smoker
Primary: RML
Metastasis: LN 7

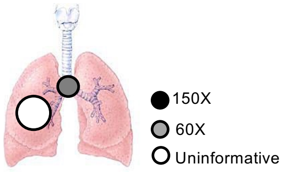

IntoGen drivers in ***Bold***
Het in germline

LN 7

APC	Splice
NOTCH1	Splice
NOTCH1	A465S
SMARCA4	M46fs
TP53	I195T
WT1	S227R

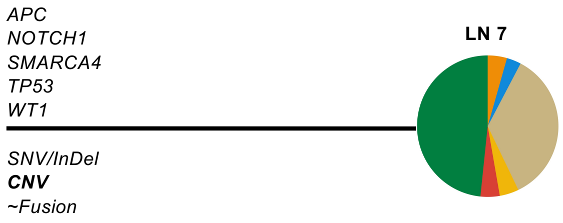

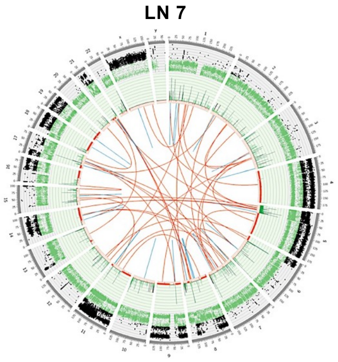

Alkylating Agents
APOBEC
Deamination 5mC
HR Defect
MMR Defect
POLE
Pol n
Smoking
Tobacco Chewing
Transcription Strand Bias T>A
Unknown

Intrachromosomal rearrangement
Interchromosomal rearrangement
CNV gain CNV loss
SNV/InDel VAF > 0.5 VAF ≤ 0.5

LUAD13
Stage IIIA
Male 61yo
Former smoker 25PY
Primary: RML
Metastasis: LN 7

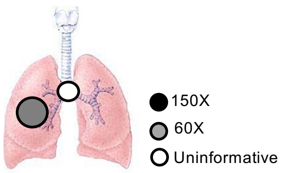

IntoGen drivers in ***Bold***
Het in germline

LN 7

AKT2	CN gain
ATM	Y1961*
AXL	CN gain
KRAS	G12C
KRAS	CN gain
MCL1	CN gain
NTRK1	CN gain
MAP4K1	CN gain
MGA	W1160*
MIR93B	CN gain
MYC	CN gain
PVT1	CN gain
TLR4	S820*

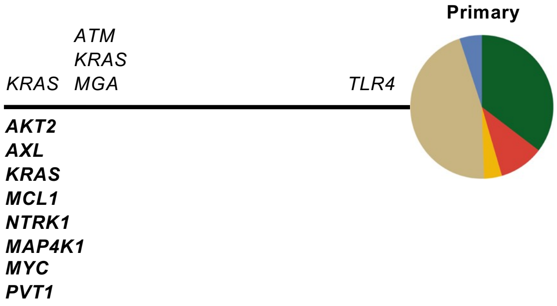

Primary

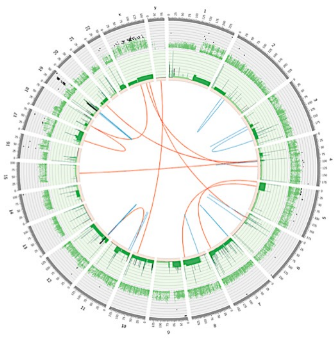

SNV/InDel
CNV
~Fusion

- Alkylating Agents
- APOBEC
- Deamination 5mC
- HR Defect
- MMR Defect
- POLE
- Pol n
- Smoking
- Tobacco Chewing
- Transcription Strand Bias T>A
- Unknown

Intrachromosomal rearrangement
Interchromosomal rearrangement
CNV gain CNV loss
SNV/InDel VAF > 0.5 VAF < 0.5

LUSC1

Stage IIIB
Male 68yo
Former smoker 45PY
Primary: LLL
Metastasis: LN 11L
Metastasis: LN 4L
Metastasis: LN 2R

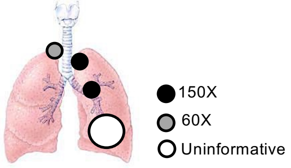

IntoGen drivers in ***Bold***
Het in germline

Private to 11L

CCND3	CN gain
FUBP1	H469fs
FGFR1	CN gain
RHEB	CN gain
SOS1	CN gain
TP63	CN gain

Shared

ERBB3	CN Gain
FGF12	CN Gain
HGF	CN Gain
IRS2	CN Gain
MAP4K3	CN Gain
MCM3	CN Gain
MECOM	CN Gain
MIR15B	CN Gain
MIR16-2	CN Gain
MLH1	V213M
MMP2	CN Gain
MYC	CN Gain
NTRK2	CN Gain
PABPC3	L558V
PCAT1	CN Gain
PVT1	CN Gain
PTEN	LOF fusion
RB1	Splice
SOX2	CN Gain
TCF4	CN Gain
TERC	CN Gain
TP53	E271*
WHSC1	LOF fusion

Private to 4L,2R

EZH2	CN gain
LAMP1	CN gain
SOS2	CN gain
STK11	K79-I84ifd

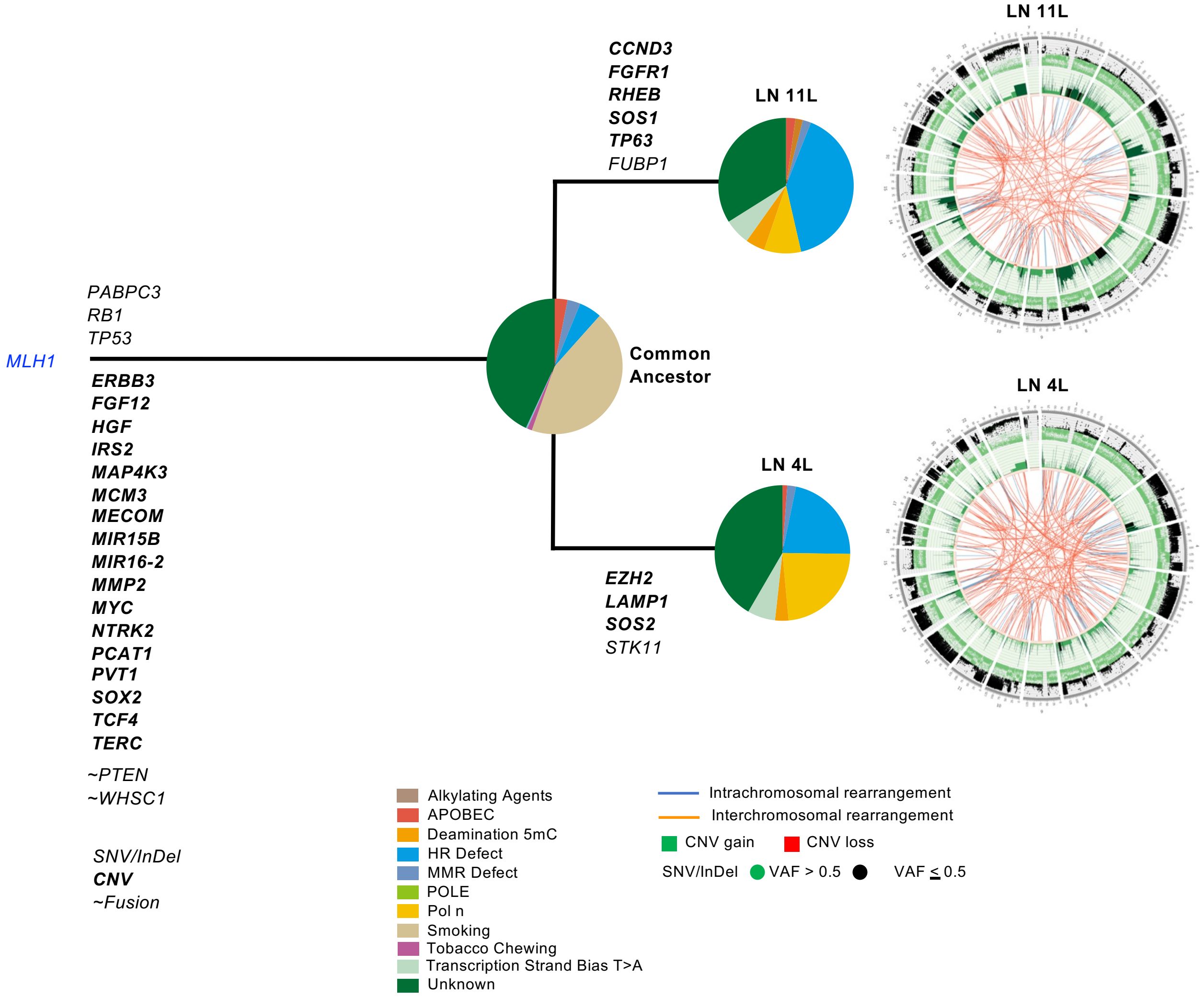

LUSC2

Female 89yo
Former smoker 15PY
Primary: LLL
Metastasis: LN 11L
Metastasis: LN 7

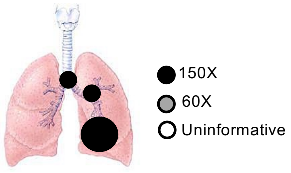

IntoGen Drivers in **Bold**
Het in germline

Shared

ARFGAP3	CN gain
ATM	LOF Fusion
CDKN2A	HozDel
CHEK1	CN gain
EP300	CN gain
ETS1	CN gain
FLI1	CN gain
FANCC	P211R
MDM2	CN gain
MUTYH	G369D
NUP107	CN gain
TP53	M160I

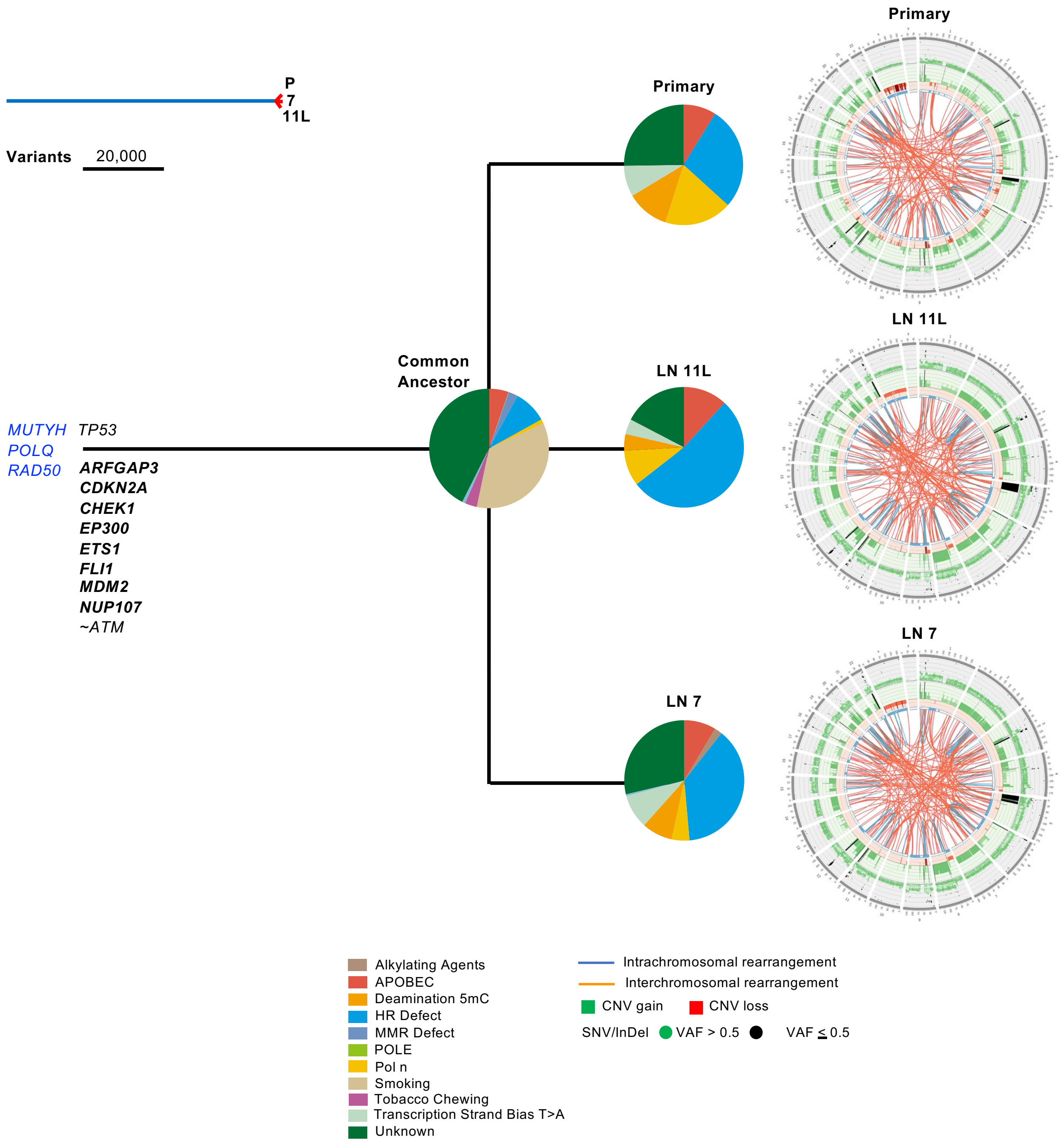

LUSC3

Stage IIIB
Male 71yo
Former smoker 20PY
Primary: RUL (P)
Metastasis: LN 4R

IntoGen drivers in **Bold**
Het in germline

Shared

MAP3K11	E454fs
ARID2	E772fs
TP53	E343*
RBM10	S219fs

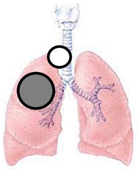

● 150X
● 60X
○ Uninformative

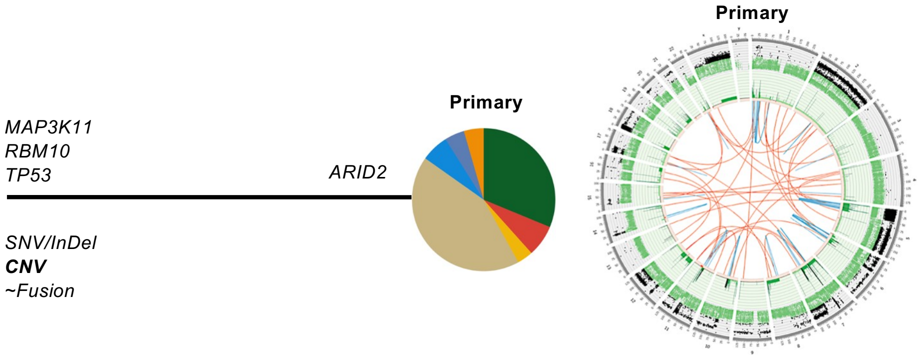

Alkylating Agents
APOBEC
Deamination 5mC
HR Defect
MMR Defect
POLE
Pol n
Smoking
Tobacco Chewing
Transcription Strand Bias T>A
Unknown

Intrachromosomal rearrangement
Interchromosomal rearrangement
CNV gain CNV loss
SNV/InDel ● VAF > 0.5 ● VAF ≤ 0.5

LUSC4

Male 78yo
Former smoker ?PY
Primary: RUL
Metastasis: LN 4R

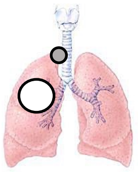

● 150X
● 60X
○ Uninformative

IntoGen drivers in **Bold**
Het in germline

LN 4R

CCND1	CN gain
ERBB4	N588K
FGF3	CN gain
FGF4	CN gain
MET	CN gain
PBRM1	E924*
PCSK6	G151R
PIK3CA	CN gain
PIK3C2B	D1307N
PLXNB2	W488*
SMARCA4	E1326K
SOX2	CN gain
TP53	H179R
TP63	CN gain

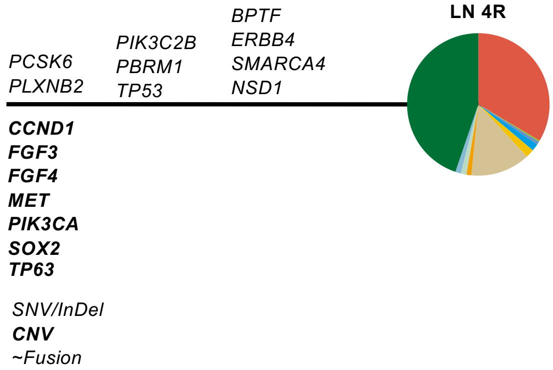

LN 4R

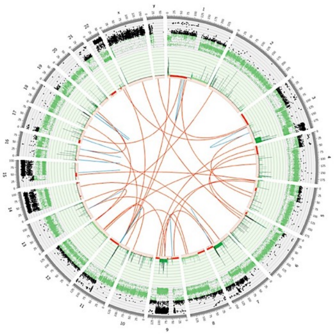

- Alkylating Agents
- APOBEC
- Deamination 5mC
- HR Defect
- MMR Defect
- POLE
- Pol n
- Smoking
- Tobacco Chewing
- Transcription Strand Bias T>A
- Unknown

Intrachromosomal rearrangement
Interchromosomal rearrangement
CNV gain CNV loss
SNV/InDel ● VAF > 0.5 ● VAF ≤ 0.5

LUSC5

Male 72yo
Former smoker 15PY
Primary: L Hilum
Metastasis: LN 7

IntoGen drivers in **Bold**
Het in germline

Primary

CDKN2A	Del
MSH3	A362H
MTAP	Del
TP53	S166*
TSC1	V200fs

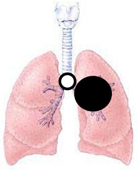

150X
60X
Uninformative

JAK2
TP53
TSC1

CDKN2A
MTAP

Primary

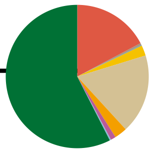

Primary

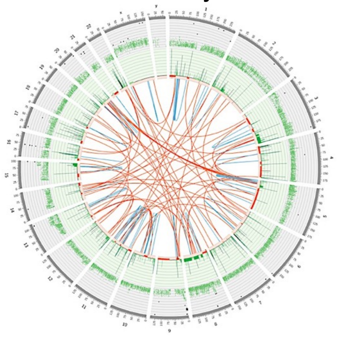

SNV/InDel
CNV
~Fusion

- Alkylating Agents
- APOBEC
- Deamination 5mC
- HR Defect
- MMR Defect
- POLE
- Pol n
- Smoking
- Tobacco Chewing
- Transcription Strand Bias T>A
- Unknown

Intrachromosomal rearrangement
Interchromosomal rearrangement
CNV gain CNV loss
SNV/InDel VAF > 0.5 VAF ≤ 0.5

SCLC1

Stage IIIA
Male 55yo
Current smoker 35PY
Primary: RML
Metastasis: LN 7

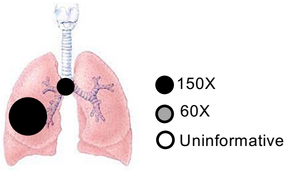

IntoGen drivers in **Bold**
Het in germline

Private to Primary		Shared		Private to LN 7	
NOTCH2	L1819S	***ACVR1B***	CN gain	*MIR99A*	CN gain
		AURKA	CN gain	*MIR125B2*	CN gain
		BRCA2	P153S	***RUNX1***	CN gain
		CREBBP	I1483F	*ATE1*-***ERG***	Fusion
		FGFR2	CN Gain		
		MYB	CN Gain		
		NFAT5	D720V		
		NTRK1	CN Gain		
		RB1	Del		
		RTEL1	T615I		
		TCF4	CN Gain		
		TP53	M160I		
		USP6	S1009F		

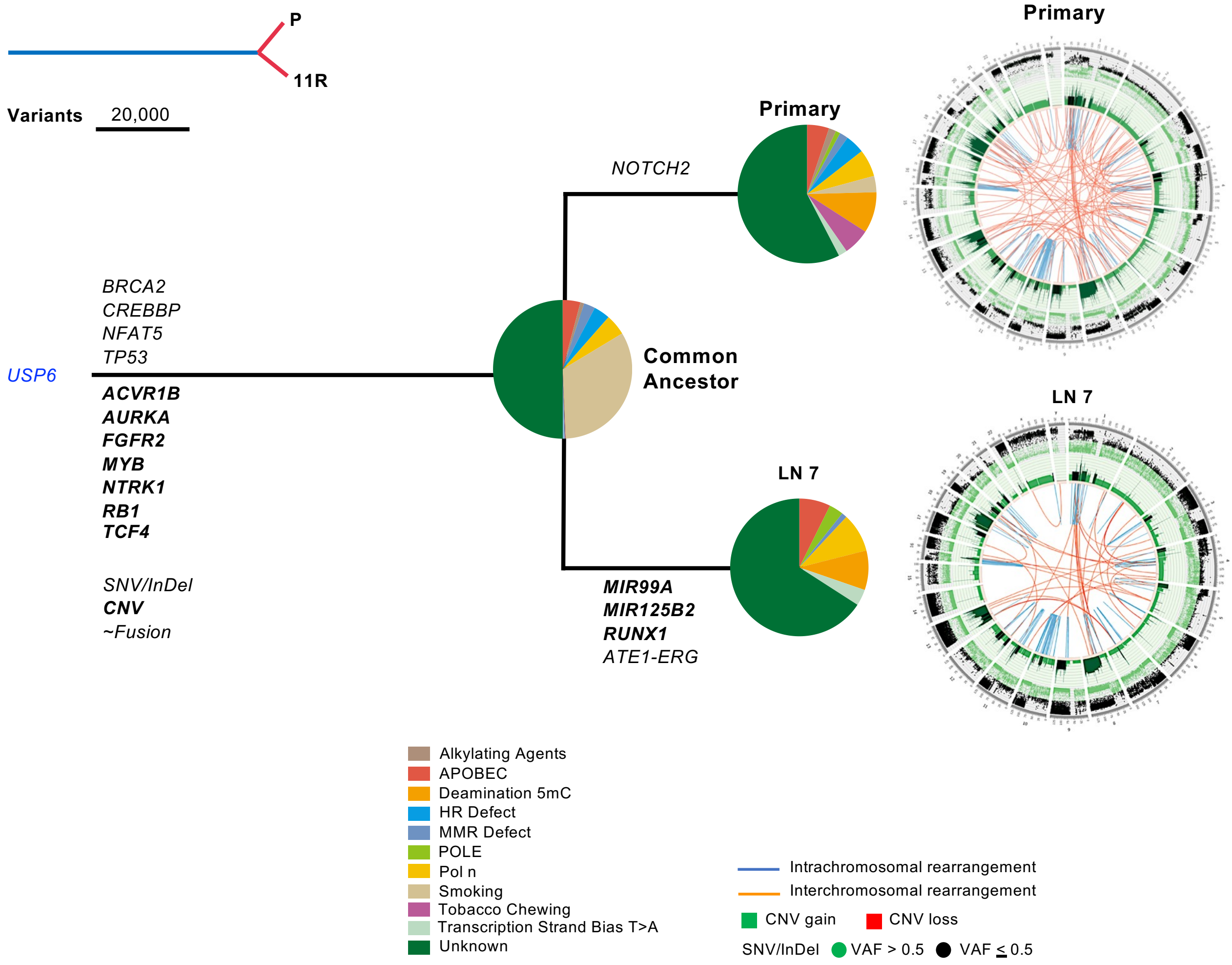

SCLC2
Stage IV
Female 48yo
Former smoker
Primary: R Hilum
Metastasis: LN 4R

IntoGen drivers in **Bold**
Het in germline

Shared

FGFR2	K568E
PTEN	Splice
POLD1	R978G
RB1	Splice
RECQL4	P103L
TOP3A	Splice
TP53	C238F
TP53BP1	Splice

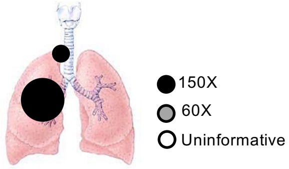

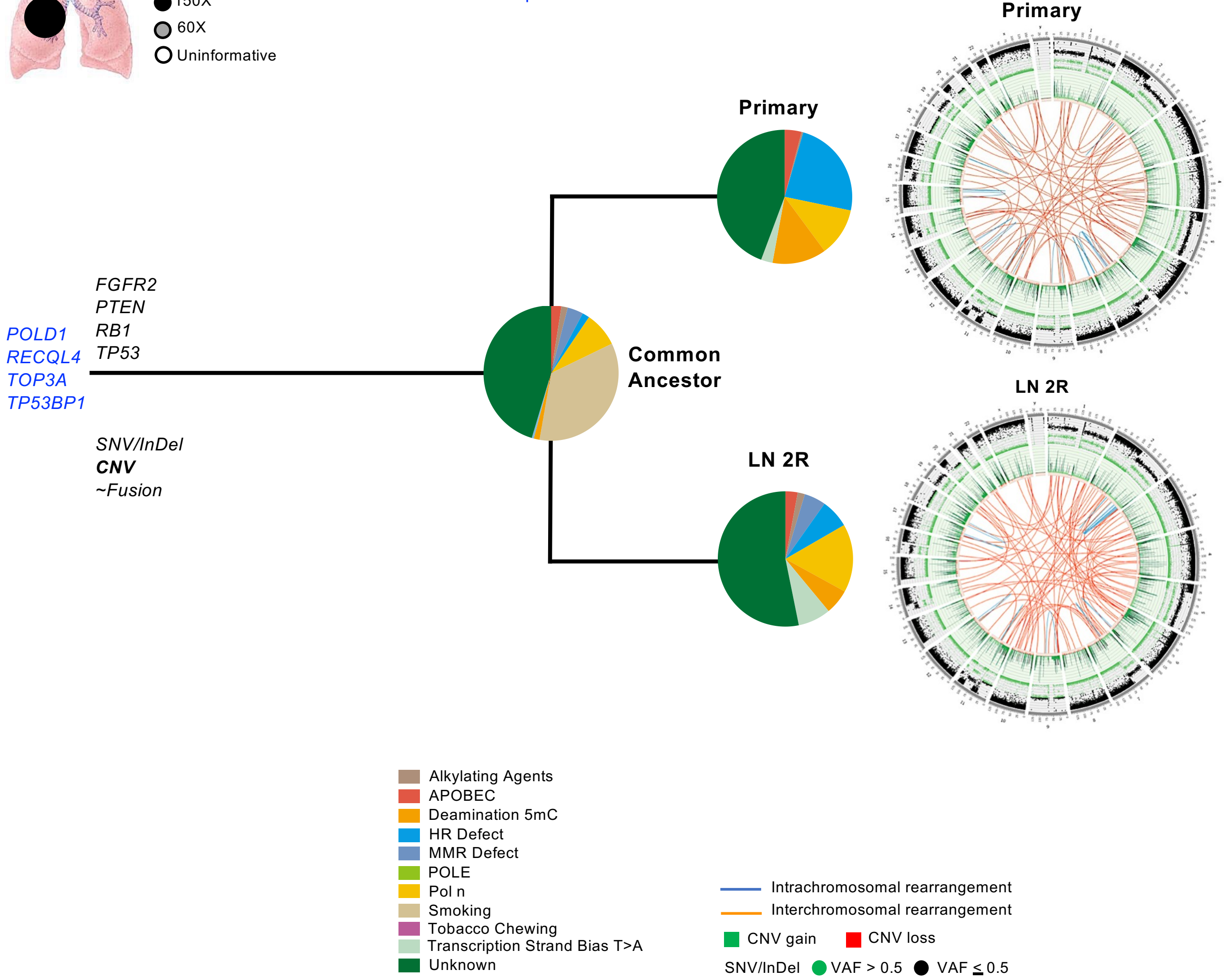

SUPPLEMENTARY TABLES

Table S1. Somatic loss-of-function mutations in non-driver genes with potential functional significance. All events associated with somatic loss-of-heterozygosity. Predictions: POLY, Polyphen; PRO, Provean; DANN, DANN Score. D, damaging; PD, potentially damaging; T, tolerated.

Gene	Variant	Case	Function	Potential Impact	POLY	SIFT	PROV	DANN
ATP7B	S882F	LUAD1	Wilson's Disease Cu^{2+} exporter	Platinum Sensitivity	PD	T	D	0.9853
ERAP2	P364L	LUAD1	Promotes MHC Class I antigen presentation	Resistance to immunotherapy	PD	D	D	0.9987
POLQ	E2016A	LUAD1	Participates in DNA replication and repair	Genomic instability, chemosensitivity	T	D	D	0.9825
WDFY3	L1058P	LUAD1	PIP3 Golgi-related protein	Defective autophagy	PD	D	D	0.9991
DPYD	A664T	LUAD3	Dihydropyrimidine dehydrogenase	Sensitivity to 5-fluorourcil	PD	D	D	0.9991
RANBP2	E2903*	LUAD3	Nuclear envelope protein, regulates TopoII	Mitotic genomic instability	.	.	.	.
FANCD2	R735Q	LUAD4	Homologous recombination of DNA double strand breaks	Chromosomal instability	PD	D	T	0.9996
PTPRD	Q1288K	LUAD4	Protein tyrosine phosphatase receptor, inhibits STAT3	Activation of STAT3 pathway	PD	D	D	0.9912
CDK5RAP2	L1455F	LUAD11	Regulates CD5 activity and mitotic spindle checkpoint	Chromosomal instability	PD	D	D	0.9989
TLR4	S820*	LUAD13	Toll-like receptor, innate immune response	Resistance to immune attack	.	.	.	.
DUSP4	V276M	LUSC3	Dual Specificity Phosphatase 4	Inactivates ERK1, ERK2 and JNK	.	PD	D	0.9989
ERCC5	E625*	LUSC3	ERCC excision repair protein	Genomic instability, platinum sensitivity	.	.	.	.
SASH1	E322*	LUSC3	Scaffold protein involved in the TLR4 signaling pathway	Mutated in an inherited skin cancer	.	.	.	.
SETBP1	T1006*	LUSC3	SET domain protein, histone lysine methyltransferase	Candidate tumor suppressor	.	.	.	.
NFAT5	D720V	SCLC1	Transcription factor, represses WNT pathway	Activation of WNT signaling	PD	PD	T	0.9857
RTEL1	T615I	SCLC1	Maintains telomere length	Chromosomal instability	T	D	D	0.9954

Table S2. Novel fusion events. GOF, gain-of-function; LOF, loss-of-function; HR, homologous recombination; OS, osteosarcoma, HNSCC, head and neck squamous cell carcinoma.

**Driver Genes**				
Event	***Case***	***Consequence***	***Impact***	***Functional implication***
3p14.3-*ATM*	LUSC2	Truncation	LOF	HR DNA repair defect, chromosomal instability, platinum sensitivity
ATE1-ERG	SCLC1	Fusion protein	GOF	Aberrant ERG-dependent transcription
DCDC2-PTEN	LUSC1	Nonsense transcript	LOF	Inactivation of the PTEN tumor suppressor
DHX57-SOS1	LUAD7	Fusion protein	GOF	Activation of the SOS1 proto-oncogene
WHSC1-11p13	LUSC1	Stop lost	LOF	Inactivation of the WHSC1, a SET H3K27 methyltransferase

**Non-Driver Genes**				
Event	***Case***	***Consequence***	***Impact***	***Functional implication***
BOD1-18q21.33	LUAD1	Stop lost	LOF	Mitotic spindle defects, chromosomal instability
DLG2-8q24.21	LUAD2	Stop lost	LOF	Candidate tumor suppressor in OS, HNSCC
2q23.1-*KDM3B*	LUAD7	Truncation	LOF	Loss of H3K9 demethylase activity
2q23.1-*MBD2*	LUAD7	Frameshift	LOF	Disruption of methylation-dependent chromatin regulation
NLRC3-18q22.1	LUAD1	Stop lost	LOF	Deregulation of innate immune signaling
14q-*POLI*	LUAD7	Truncation	LOF	Disruption of trans-lesional DNA synthesis
6p22.3-*RBL1*	LUAD7	Truncation	LOF	Deregulation of E2F transcription

Table S3. Germline loss-of-function variants in DNA repair genes associated with tumor loss-of-heterozygosity. BER, base excision repair; DSBR, double strand break repair; HR, homologous recombination; MMR, mismatch repair; MMEJ, microhomology mediated end joining; ExAC, allele frequency in the ExAC database; NHEJ, non-homologous end-joining; OxDR, oxidative DNA damage response; CI, conflicting interpretations of pathogenicity; LP, likely pathogenic; P, pathogenic; US, uncertain significance.

Gene	Variant	Case	Function	Potential Impact	ExAC	ClinVar	DANN Score	Varsome
ATM	L1420F	LUAD3	HR	Chromosomal instability	0.012	CI	0.9931	US
ATR	H160R	LUAD4	HR	Chromosomal instability	0.00006	.	0.9850	US
CHEK1	R36*	LUAD1	Checkpoint kinase	DNA damage, chromosomal instability	0	.	.	.
ERCC8	A219P	SCLC1	BER	Tobacco mutagenesis, chromosomal instability	0	.	0.9964	US
EXO1	G264R	LUAD1	MMR	Hypermutation	0	.	0.9994	US
FANCA	S858R	LUSC1	HR, crosslink repair	Chromosomal instability	0.01	CI	0.945	LP
FANCC	P211R	LUSC2	HR	Chromosomal instability	0	CI	0.9977	US
FANCL	P17R	LUAD2	HR	Chromosomal instability	0.0008	.	0.9979	US
LIG4	A842D	LUAD9	NHEJ	Tobacco mutagenesis, chromosomal instability	0.0025	US	0.9993	US
MBD4	N467S	LUAD2	MMR	Sensitivity to 5FU	0.0019	.	0.9971	US
MUTYH	G369D	LUSC2	MMR, OxDR	Tobacco mutagenesis, hypermutation	0.0001	LP	0.9985	P
MUTYH	V207M	LUAD3	MMR, OxDR	Tobacco mutagenesis, hypermutation	0.006	US	0.9988	US
NEIL1	A106fs	LUAD12	BER, OxDR	Tobacco mutagenesis, hypermutation	0	.	.	.
NEIL1	Splice	LUAD11	BER, OxDR	Tobacco mutagenesis, hypermutation	0.008	.		.
POLB	R89W	LUAD12	BER	Tobacco mutagenesis, hypermutation	0.00008	.	0.9993	US
POLD1	R978G	LUAD13	Lagging strand synthesis	Hypermutation	0	.	0.9923	US
POLE	I1127F	LUAD7	DNA repair	Hypermutation	0	.	0.9887	US
POLE	G2092A	LUAD11	DNA repair	Hypermutation	0	.	0.9982	US
POLQ	L1720F	LUSC2	MMEJ, NHEJ	Chromosomal instability	0.0019	.	0.9867	US
POLQ	Y2420C	LUSC5	MMEJ, NHEJ	Chromosomal instability	0.0003	.	0.9978	US
POLQ	R438W	SCLC2	MMEJ, NHEJ	Chromosomal instability	0.00008	.	0.9994	US
PRKDC	A609V	LUAD2	NHEJ	Chromosomal instability	0	.	0.9979	US
PRKDC	L1706Q	LUAD3	NHEJ	Chromosomal instability	0.00197	CI	0.9925	US
RAD50	R1279C	LUAD5	HR	Chromosomal instability	0	US	0.9994	US
RAD50	R884H	LUAD6	HR	Chromosomal instability	0	CI	0.9991	US
RAD51B	K243R	LUAD3	HR	Chromosomal instability	0.01073	.	0.9989	US
RECQL4	P103L	SCLC1	DNA helicase	Tobacco mutagenesis, chromosomal instability	0.0006	US	0.9866	US
REV1	K104R	LUSC1	TLS	Tobacco mutagenesis	0.0003	.	0.9949	US
RPA1	R389W	LUAD2	Replication stress response	Chromosomal instability	0.0010	.	0.9993	US
TDG	I363fs	LUAD12	MMR, deamination repair	Tobacco mutagenesis, hypermutation	0	.	.	.

Table S4. Characteristics of TCGA patients. NOS, not otherwise specified.

Sex	Male	360
	Female	216
Histology	Acinar adenocarcinoma	6
	Adenocarcinoma mixed	48
	Adenocarcinoma NOS	154
	Basaloid squamous cell carcinoma	9
	Bronchoalveolar carcinoma	10
	Clear cell adenocarcinoma	1
	Micropapillary adenocarcinoma	1
	Mucinous adenocarcinoma	1
	Mucinous colloid carcinoma	3
	Papillary adenocarcinoma	9
	Papillary squamous cell carcinoma	3
	Squamous cell carcinoma NOS	321

DESCRIPTION OF ADDITIONAL SUPPLEMENTARY FILES

Data File S1. Pan-cancer and lung cancer driver genes.

Data File S2: Annotated list of DNA repair genes.

Data File S3. Details of all somatic variants in all samples.

Data File S4. Details of germline variants in DNA repair genes.

Data File S5. Results of TCGA analysis.

SUPPLEMENTARY MATERIALS AND METHODS

Primary analysis

Reads were mapped to the human reference genome hs37d5 (b37 + decoy) using bwa-mem v0.7.10-r789.[1] Alignment BAM files were sorted and duplicate reads were marked using Novosort v1.03.01 (Novocraft Technologies). Multiple sequencing lanes comprising the tumor samples were merged using Novosort Merge v1.03.01 (Novocraft Technologies). Reads were aligned around indels using GATK Indel Realigner v3.3-0-g37228af.[2]

Variant calling

GEMINI v0.18.3[3] was used to package variants into databases and annotate them with pathogenicity scores and population allele frequencies. We combined the SNV and Indel VCF files using GATK CombineVariants v3.3-0-g37228af.[2] We then renamed the sample IDs in the VCF to the sample names of the current normal and tumor. We added the standard VCF FORMAT tags of GT, GQ, AD and DP. Finally, we set FILTER to PASS for variants that are QSI_REF or QSI_REF where the QSS/QSI value is 15 or greater, this acts to let loss of heterozygosity variants show up in subsequent variant queries where failed variants are typically excluded. Resulting per-sample somatic VCF files were combined across the entire cohort with GATK CombineVariants v3.3-0-g37228af[2] to result in a single joint-called VCF file containing all somatic variants that are present in at least one tumor or metastatic sample. For germline variants, we sued used GATK HaplotypeCaller v3.3-0-g37228af[2], and joint-called the resulting GVCFs using GATK GenotypeGVCFs v3.3-0-g37228af[2] and recalibrated quality scores across the cohort with GATK Variant Quality Score Recalibrator v3.3-0-g37228af[2]. This resulted in a single joint-called VCF file containing all germline variants that are present in at least one sample. All reported variants were manually validated using IGV.[4]

TCGA data set

Patients from TCGA LUAD and LUSC datasets[5,6] were selected based upon documented smoking history, and no record of previous malignancy or neoadjuvant therapy. Patients with tumors with *EGFR* mutations or *EML-ALK* fusions were excluded. Patients with multiple tumor samples were considered as one case and the tumor sample randomly selected. Data were obtained from TCGA as BAM files and variants were called and processed using the same approach as for the EBUS-TBNA data.

Somatic signatures

Somatic signatures were determined using somatic variants identified by Strelka. We used Strelka default PASS variants only, which enriches for real somatic variants by excluding variants present in the germline and variants with insufficient evidence of somatic status, and was then used to process the variants and calculate motif matrices of the 96 possible mutation contexts. These matrices were compared against the 30 COSMIC mutational signature profiles identified as part of The Cancer Genome Atlas[7] to determine loadings, fits and residuals using the non-negative least squares algorithm implemented in the nnls R package (cran.r-project.org/web/packages/nnls/). Resulting profiles were visualised with custom ggplot2[8] plots to show the observed mutational signature profile, the fit against the 30 COSMIC profiles[9], the differences (residuals) between the two and an overall percentage match of the observed profile to each of the 30 COSMIC profiles.

Somatic copy number variant calling and analysis

Sequenza applies an algorithmic approach to estimate overall tumor cellularity which we used to determine whether samples should be sequenced to a greater depth. It also produces segments for each copy number change through the genome, including the ploidy of the A and B allele for each segment. Analysis was performed to identify all segments that overlapped by 1 or more bases with any of the genes in the consensus cancer driver gene list described above. All reported variants were manually validated using IGV.[4]

Copy number variation
To compare copy number profiles between samples, events smaller than 10kb were excluded on the basis that they did not validate in IGV. To minimise noise, for each sample we averaged the copy number values across cytoband coordinates in proportion to the number of bases covered by each variant in the cytoband, assuming diploid status for coordinates without variants. This resulted in a profile of copy number values for each of the 811 autosomal cytobands per sample.
To compare genome-wide CNV profile changes, we calculated pairwise coefficients of determination (R^2) between all samples and generated heatmaps using the heatmap.2 function from the gplots package for R. To visually display profiles across all samples for each patient, we generated segmented data files (*.seg) containing cytoband coordinates and copy number values and used IGV to plot these inline and scaled per patient. All reported variants were manually validated using IGV.[4]

Phylogenetic reconstruction.
Accurate phylogenetic reconstruction requires high confidence in shared and private events. To achieve this for our multi-region cases, we first used GATK CombineVariants v3.3-0-g37228af[2] on all short somatic variants per patient to determine all variants present in one or more samples. We then ran Strelka again for each sample and forced it to call variants at every position where a variant was present in any sample from the patient. Finally, we combined the re-called variants to obtain genotype and sequencing depth information for each sample, even when there was initially not enough evidence for denoting somatic status.

We proceeded to exclude variants deemed to have insufficient quality or information to enable determining shared or private status. We applied exclusion regions using BEDTools v2.22.0[10] to discard variants called in difficult to sequence regions of the genome. Variants were excluded where they did not pass default Strelka quality thresholds in at least one sample. We also excluded variants where no information was obtained for at least one sample during the re-calling, multi-allelic variants and those outside the autosome. To ensure we only analyzed somatic events, we discarded all variants where the germline had one or more reads supporting the alternate allele. To ensure adequate sequencing coverage, we discarded all variants where any one of the somatic samples had an overall depth of below 50. To ensure sufficient evidence of somatic status we excluded variants where no sample had 6 or more high quality reads supporting the alternate allele. Finally, to control for cellularity differences, a sample needed at least 2 reads supporting the alt allele to be classed as sharing a variant.

Visualization
Visualization was performed using Circos v0.69.[11]

SUPPLEMENTARY REFERENCES

1 Li H, Durbin R. Fast and accurate short read alignment with Burrows-Wheeler transform. *Bioinformatics* 2009; **25**: 1754–1760.

2 McKenna A, Hanna M, Banks E, Sivachenko A, Cibulskis K, Kernytsky A *et al.* The Genome Analysis Toolkit: a MapReduce framework for analyzing next-generation DNA sequencing data. *Genome Res* 2010; **20**: 1297–1303.

3 Paila U, Chapman BA, Kirchner R, Quinlan AR. GEMINI: integrative exploration of genetic variation and genome annotations. *PLoS Comput Biol* 2013; **9**: e1003153.

4 Robinson JT, Thorvaldsdottir H, Winckler W, Guttman M, Lander ES, Getz G *et al.* Integrative genomics viewer. *Nat Biotech* 2011; **29**: 24–26.

5 Cancer Genome Atlas Research Network, Campbell JD, Brooks AN, Berger AH, Lee W, Chmielecki J *et al.* Comprehensive molecular profiling of lung adenocarcinoma. *Nature* 2014; **511**: 543–550.

6 Cancer Genome Atlas Research Network, Lawrence MS, Voet D, Jing R, Cibulskis K, McKenna A *et al.* Comprehensive genomic characterization of squamous cell lung cancers. *Nature* 2012; **489**: 519–525.

7 Huang P-J, Chiu L-Y, Lee C-C, Yeh Y-M, Huang K-Y, Chiu C-H *et al.* mSignatureDB: a database for deciphering mutational signatures in human cancers. *Nucl Acid Res* 2018; **46**: D964–D970.

8 Wickham H. *ggplot2: Elegant Graphics for Data Analysis*. First. 2010.

9 Forbes SA, Beare D, Boutselakis H, Bamford S, Bindal N, Tate J *et al.* COSMIC: somatic cancer genetics at high-resolution. *Nucl Acid Res* 2017; **45**: D777–D783.

10 Quinlan AR, Hall IM. BEDTools: a flexible suite of utilities for comparing genomic features. *Bioinformatics* 2010; **26**: 841–842.

11 Krzywinski M, Schein J, Birol I, Connors J, Gascoyne R, Horsman D *et al.* Circos: an information aesthetic for comparative genomics. *Genome Res* 2009; **19**: 1639–1645.
